# Geographical Distribution and Selection of European Honey Bees Resistant to *Varroa destructor*

**DOI:** 10.3390/insects11120873

**Published:** 2020-12-08

**Authors:** Yves Le Conte, Marina D. Meixner, Annely Brandt, Norman L. Carreck, Cecilia Costa, Fanny Mondet, Ralph Büchler

**Affiliations:** 1 INRAE, Abeilles et Environnement, 84914 Avignon, France; fanny.mondet@inrae.fr; 2 Landesbetrieb Landwirtschaft Hessen, Bee Institute, Erlenstrasse 9, 35274 Kirchhain, Germany; marina.meixner@llh.hessen.de (M.D.M.); annely.brandt@llh.hessen.de (A.B.); ralph.buechler@llh.hessen.de (R.B.); 3 Carreck Consultancy Ltd., Woodside Cottage, Dragons Lane, Shipley RH13 8GD, West Sussex, UK; norman.carreck@btinternet.com; 4 Laboratory of Apiculture and Social Insects, University of Sussex, Falmer, Brighton BN1 9QG, East Sussex, UK; 5 CREA Research Centre for Agriculture and Environment, via di Saliceto 80, 40128 Bologna, Italy; cecilia.costa@crea.gov.it

**Keywords:** honey bee, *Varroa destructor*, resistance, breeding, selection, survival

## Abstract

**Simple Summary:**

The parasitic mite *Varroa destructor* is a major challenge to honey bee populations worldwide. Some honey bee populations are resistant to the mite, but most of the commercially used stocks are not and rely on chemical treatment. In this article, we describe known varroa-resistant populations and the mechanisms which have been identified as responsible for survival of colonies without beekeeper intervention to control the mite. We review traits that have potential in breeding programs, discuss the role played by *V. destructor* as a vector for virus infections, and the changes in mite and virus virulence which could play a role in colony resistance. We also describe results of surveys carried out (mostly within Europe) on the presence of naturally surviving honey bee populations, on the commercial availability of mite resistant stock and on the traits considered in breeding programs. We found that there is a growing interest and awareness among beekeepers, but that there are very few commercially available resistant lines; some from breeding programs and some from naturally selected populations. The most commonly considered traits for assessing varroa resistance are linked to mite reproduction and specific hygienic behavior of the bees.

**Abstract:**

Developing resistance to the varroa mite in honey bees is a major goal for apicultural science and practice, the development of selection strategies and the availability of resistant stock. Here we present an extended literature review and survey of resistant populations and selection programs in the EU and elsewhere, including expert interviews. We illustrate the practical experiences of scientists, beekeepers, and breeders in search of resistant bees. We describe numerous resistant populations surviving without acaricide treatments, most of which developed under natural infestation pressure. Their common characteristics: reduced brood development; limited mite population growth; and low mite reproduction, may cause conflict with the interests of commercial beekeeping. Since environmental factors affect varroa mite resistance, particular honey bee strains must be evaluated under different local conditions and colony management. The resistance traits of grooming, hygienic behavior and mite reproduction, together with simple testing of mite population development and colony survival, are significant in recent selection programs. Advanced breeding techniques and genetic and physiological selection tools will be essential in the future. Despite huge demand, there is no well-established market for resistant stock in Europe. Moreover, reliable experience or experimental evidence regarding the resistance of stocks under different environmental and management conditions is still lacking.

## 1. Introduction

Varroa and honey bees have been a subject of growing interest since the mite invaded the world. In the past five years, several reviews about varroa and honey bees have been published. For example, Mondet et al. 2020 published a review on honey bee survival mechanisms against the parasite *V. destructor* with a focus on genomics research effort. The specificity of our review is the focus on known varroa-resistant populations and the mechanisms that have been identified to be responsible for survival of colonies with or without beekeeper intervention to control the mite. A second focus of this paper introduces the results of two surveys: one on the presence of naturally surviving honey bee populations in European countries; and one on the commercial availability of mite resistant stock and on the traits considered in breeding programs. 

## 2. Review of Scientific Research

### 2.1. Why Should We Select Bees Resistant to the Mite?

The parasitic mite *Varroa destructor* spread throughout Europe in the second half of the last century, and now represents the greatest problem for the Western honey bee, *Apis mellifera*. Since then, the beekeeping industry and hobby beekeepers have had to face a major challenge. The regular use of chemical treatments to control the mite has several disadvantages such as high costs and labor, residues in bee products, and the rapid emergence of mite populations resistant to acaricides [1]. Consequently, there is an urgent need to use alternative control methods for the mite. Using mite-resistant honey bees is generally agreed to be the most sustainable way to proceed. Research into varroa resistance in honey bees started in the 1980s and continues to receive a large amount of scientific interest and practical attention in Europe and worldwide [2,3,4,5]. There are several different approaches to obtaining varroa resistant bees.

One approach is to consider that some honey bee populations living with the mite for many generations without varroa control will naturally develop resistance that will contribute to an equilibrium between the parasite and its host. This has been demonstrated with the original host of *Varroa* spp., the Eastern honey bee *Apis cerana*, and with *A. m. scutellata* in southern Africa or imported to South America as the Africanized bee, which has a large degree of resistance to the mite. In Europe and the USA, a few small populations of European strains have been found to be naturally resistant to the mite. The original host, *A. cerana*, has co-evolved with its host over millions of years, which is not the case in *A. mellifera*. However, the presence and survival of these populations demonstrates that a period of less than 100 years can be enough to build up an equilibrium between the host and the parasite.

While naturally resistant strains mainly consist of feral colonies with no impact of the beekeeper, another approach can be mass selection using a large group of varroa infested honey bee colonies, which are allowed to live with the mite without any treatment and either die or survive. This has been called the “Bond test” (“Live and let die!”), and was developed in Sweden and in France and then in a few other European countries [6]. 

Finally, a more academic approach for artificial selection is to develop a genetic selection based on chosen phenotypic characters and quantitative genetic tools. This approach has been utilized more or less successfully since the 1980s by several different research teams around the world.

### 2.2. Naturally Selected Populations and Their Known Mechanisms

Apart from *Apis cerana*, which is the original host, there are few cases of naturally varroa resistant populations in *Apis mellifera* subspecies. 

#### 2.2.1. *Apis cerana*

To date, four species of the genus *Varroa* are known-*V. jacobsoni*, *V. rindereri*, *V. underwoodi*, and *V. destructor*. All of these mites are brood parasites of cavity nesting Asian honey bees, mainly *Apis cerana* but also *A. koschevnikovi*, *A. nigrocincta* and *A. nuluensis* [7]. The natural distribution range of these species nowhere overlaps with that of *A. mellifera*, the Western honey bee native to Europe. Only after humans introduced European *A. mellifera* to far eastern Asia for the purpose of honey production, were varroa mites able to shift host and establish themselves on *A. mellifera*, where it has been known since at least 1915 [8]. At that time and later, when varroa reached Europe and other continents, it was thought that the species in question was *Varroa jacobsoni*, the first *Varroa* spp. mite described from Indonesia [9]. Only in 2000 did it become clear that the most detrimental parasite of apiculture in fact belonged to a different and previously undescribed species, *V. destructor*, native to *A. cerana* in northeast Asia [10]. The Eastern honey bee *Apis cerana*, the original host of *Varroa* spp., tolerates infestation without suffering serious damage. Several mechanisms enable it to do this, all of which operate to limit mite population development. Firstly, and most importantly, the mites reproduce successfully mostly in the drone brood of *A. cerana*. This means that mite reproduction can only take place during the relatively short period when drone brood is present. Furthermore, in cases where multiple infestation of a drone cell occurs, and the pupa dies, perhaps due to viral infection, the worker bees do not uncap the cell, thus entombing the dead pupa and the mites which cannot thereby continue their cycle, and sealing off infectious agents [11,12]. Should the mites enter worker cells, adult bees can detect them and uncap the cells before the mites can reproduce. The bees open the cell, remove the mite and reseal it, allowing the pupa to develop normally [13].

Hänel and Koeniger [14] suggested that bee Juvenile Hormone III was the stimulus for *Varroa* spp. mites to lay eggs, and that the observed differences in hormone titers between *A. cerana* and *A. mellifera* could explain the inability of mites to reproduce in worker brood of *A. cerana*, but this was later disputed by Rosenkranz et al. [15].

In contradiction to these previous reports, two recently published studies [16,17] showed that *A. cerana* worker larvae are not only as attractive to *V. destructor* mites as drone larvae, but also that reproduction inside the worker cell is initiated and can be successfully completed if the larval development is normal. In *A. cerana* colonies, however, a high proportion of infested worker larvae show abnormal development, thereby triggering a behavior of the adult workers analogous to the Varroa Sensitive Hygienic behavior (VSH) described in *A. mellifera* and preventing mite population growth from reproduction in worker brood.

Workers of *A. cerana* also show several forms of grooming behavior whereby mites are removed from other workers and damaged, often by the removal of legs [18]. This has been demonstrated in several studies, although Tewarson et al. (1992) [19] and Rosenkranz et al. (1993) [13] have suggested that the behavior observed may have at least partly been induced by the experimental techniques involving the transfer of mites from one colony to another, and may not relate to the normal situation in colonies. The quantitative contribution of grooming to mite resistance in *A. cerana* is still not clear and may be very limited [11,20]. 

Colonies of *A. mellifera* were introduced to Japan in 1876 and no doubt to other parts of the Far East in the same period, and kept alongside *A. cerana*. It seems curious, therefore, that reports of the devastating damage caused by the mite did not occur until many years later when the mite was found in Europe in the early 1970s. How could it have existed in *A. mellifera* colonies in the Far East for sixty years without reports of widespread damage when in Europe colony death commonly occurs less than three years after infestation?

#### 2.2.2. Africanized Honey Bees

The predominant honey bee of southern Africa is *A. m. scutellata*. In the 1950s, *A. m. scutellata* queens from South Africa were experimentally introduced to Brazil, as it was felt that, being tropical bees, they would be more suitable for the tropical conditions there than the European strains previously used. Some swarms accidentally escaped, and the bees indeed proved to be well adapted, rapidly spreading throughout the continent, into Central America and now into the southern USA. The aggressive behavior of these “Africanized” bees resulted in a fearsome reputation. Varroa mites arrived in South America already in 1971 [21], but are not viewed as a serious problem by beekeepers using Africanized bees (AHB) in Brazil. Few colonies are treated, yet mite populations remain small. 

The work of Anderson and Trueman (2000) [10] demonstrated not only that “*Varroa jacobsoni*” was in fact several species, but that there were several different variants or haplotypes of *V. destructor*, notably the Korean haplotype, which seems very virulent, and the Japan/Thailand haplotype, which seems less virulent. Due to different invasion events, the Japan/Thailand haplotype became established in South America, whilst the Korean haplotype spread throughout Europe and USA. The reduced fertility of the Japan/Thailand haplotype was initially believed to be the main reason why Africanized bees (AHB) were resistant to varroa [22]. However, during the past 20 years in Brazil, the original Japan/Thailand haplotype has been replaced by the more virulent Korean haplotype, and there has been a corresponding increase in both the mite fertility (from 35% to 72%) and the number of mites producing at least one viable offspring in worker brood (from 56% to 80%) [23]. Despite this dramatic increase in the mite’s reproductive ability, however, infestation levels remain low and AHB remain varroa tolerant with high hygienic behavior [24]. This also supports findings from Mexico where AHB have long been known to be varroa tolerant despite the presence of the Korean haplotype. This suggests that several resistance mechanisms are at work in the Africanized bees. Recent findings suggest that the tolerance of Africanized bees to *V. destructor* in Mexico is related to adult bee mechanisms such as, for example, hygienic and grooming behavior [25].

The wax brood cells made by *A. m. scutellata* are slightly smaller than those made by European strains. Message and Gonçalves (1995) [26] found that this led to a reduction in the infestation rate. They suggested that larger larvae, reared in larger cells, received more visits from infested nurse bees than those in small cells, increasing the opportunity for mites to invade, and therefore the likelihood of infestation.

Ritter and De Jong (1984) [27] found bees of *A. m. scutellata* to have a post-capping stage duration (PSD) significantly shorter than that of European strains in Germany, and suggested that this may explain the lower rate of infestation. Conversely, in more extensive studies in Mexico, Vandame et al. (1998) [28] found no significant differences in the PSD of experimental colonies of *A. m. scutellata* and European strains. The observed differences therefore may represent the large natural variability in post capping times at different times of the year and under different climatic conditions.

Worker brood from European strains has also been found to be twice as attractive to mites than that of *A. m. scutellata* [29,30].

Grooming behavior has been suggested as a possible tolerance mechanism in *A. m. scutellata*, and various studies have observed grooming behavior, although to a lesser degree than in *A. cerana*. Vandame et al. (1998) and others [28] have concluded that grooming is unlikely to be a significant factor in limiting mite population growth. Several studies have also noted removal of infested brood in *A. m. scutellata* colonies, but mites are not removed from the colony and have subsequently been observed to enter brood cells and breed again. Vandame et al. (1998) [28] concluded, however, that the reproductive potential of these removed mites is reduced. Conversely, several peer-reviewed studies have shown that the most important and the only statistically significant factor associated to low Varroa population growth and mite removal is grooming behavior, demonstrating that grooming behavior can be important in restraining mite population growth in Africanized bees. [31,32,33,34].

In most studies, the ability of *A. m. scutellata* to tolerate mites seems to be associated with the degree to which the mites fail to lay eggs or to produce viable offspring [22]. Ritter and De Jong (1984) [27] found that only 43% of female mites produced offspring when infesting *A. m. scutellata* in Brazil as compared to 76% in European bees in Germany.

#### 2.2.3. African Honey Bees

While Africanized bees in South America and their resistance mechanisms towards varroa mites have long been in the focus of scientific interest, the fate of bees on the African continent for a long time received very little attention. *V. destructor* presence has been confirmed since about 1990 in North Africa [35]; it was found in South Africa in 1997 [36], and since then has been documented in various countries of west and east Africa. According to the most recent overview [37], the mite is now known to occur in 31 African countries, but there are still several regions with no data available, so this is likely to be an underestimation.

In contrast to the situation in Europe or North America, beekeeping in Africa relies on constant influx from the huge reservoir of wild colonies that have been estimated at 310 million [38]. Beekeeping operations, even large commercial ones, acquire their colonies mostly by trapping swarms, and deliberate breeding is nearly absent [37,38].

However, infestation with varroa mites does not seem to lead to colony losses, nor to greatly negatively affect bee colonies [39,40,41,42]. It has been reported that, in South Africa, resistance to varroa developed within six to seven years after first invasion [41], but few data are available from other parts of the continent. Various characters have been described that contribute to the resistance against mites, for instance: the short post-capping stage, especially in *A. m. capensis* [43]; enhanced grooming behavior [44]; and the removal of mites through hygienic behavior [45] and recapping behavior [46]. In *A. m. scutellata* colonies, survival has been attributed to reduced varroa population growth [41] and the low prevalence of viruses [40]. In addition, absconding, a specific type of swarming behavior, typical for tropical bees, may contribute to significantly reducing mite infestation levels. When a colony absconds, all adult bees and the queen leave the hive suddenly, leaving behind all brood. This behavior occurs when the colony is disturbed, if it is diseased or in conditions of lack of food, for example in periods of drought. In the case of high mite infestation, an absconding colony will thus get rid of all reproducing mites trapped in the brood nest. In Ethiopia, the demonstrated resistance of *A. m. simensis* is partly explained by low brood infestation levels, low capability of producing reproductive progeny, as well as high failure to produce adult male progeny [47].

#### 2.2.4. *A. m. capensis*

Workers of *A. m. capensis*, the Cape honey bee found in the extreme south west of South Africa, have a development time in the sealed cell (PSD) nearly one day shorter than that of European subspecies [43]. This is sufficient to ensure that the third mite offspring (i.e., the second daughter mite) does not reach the adult stage before the worker bee emerges. Together with the proportion of mites that do not produce fertile offspring, which appears to be greater than in European strains, this contributes to a low mite population increase in the worker brood [48]. Researchers found that hybrid bees between *A. m. capensis* and *A. m. carnica* showed the same reduced development time. *A. m. capensis* is, however, unsuitable for beekeeping in Europe for several reasons. For instance, when introduced into colonies of other *A. mellifera* subspecies, such as in a hybrid zone with *A. m. scutellata* in South Africa, it becomes a social parasite, producing “pseudoqueens” which take over the colony. *A. m. capensis* is slightly larger than other African bees, and the amount of room in the brood cell can affect the reproductive success of the mite in some extreme cases. When the oversized parasitic *A. m. capensis* are reared in *A. m. scutellata* colonies, they fill the entire cell, thus preventing the male varroa mite from reaching the feeding site on the abdomen of the pupa [49], so all the female offspring cannot mate and remain infertile. Despite this discovery, however, reducing the cell size of the comb has so far failed as a varroa control mechanism because it is not the size per se, but the amount of space within the cell that is important.

#### 2.2.5. Surviving Honey Bees Populations from France

Varroa mites invaded France in the 1980s, and most wild and untreated colonies were killed by the mites within two years. A first observation of naturally occurring varroa surviving bee colonies (VSB) was made in 1994 in the west of France, near Le Mans, where wild and untreated colonies seemed to survive the mite infestation for a few years. In 1999, 10 out of 12 of such untreated colonies were still surviving. Then, 82 colonies that were untreated for at least two years were collected in two apiaries, one in the North of France (Le Mans) and one in the South (Avignon) to characterize their survival without varroa control. These colonies were managed only for their survival. They were allowed to swarm and to naturally replace their queens. On average, the survival of those colonies was 7.88 ± 0.3 years, with a maximum of 15 years [50].

Varroa populations were estimated by counting natural mite mortality using a screened bottom board to collect the mites [7,51]. The number of mites collected in the VSB was three times lower than in varroa-susceptible control colonies [50] and continued to be lower all year round, suggesting that VSB have developed resistance mechanisms to inhibit the growth of varroa populations. 

Various hypotheses have been tested to explain this phenomenon. Martin et al. (2002) [49] showed that the VSB have a better ability to recognize the mites compared to control bees. Thus, VSB could be more efficient in their ability to detect and get rid of the mites on workers through grooming behavior, similar to the behavior observed on *Apis cerana* [18]. Observations show that VSB are also able to detect and remove mite-infested pupae from their cells (Anderson and Le Conte, personal observations). It was confirmed later that the mites in the Avignon population had high levels of infertility and that they show suppressed mite reproduction (SMR) [52]. Recent results have shown the same for the VSB from Le Mans. Interestingly, gene expression analysis of the VSB shows over-expression of a set of genes related to responsiveness to olfactory stimuli compared with varroa susceptible bee colonies [53].

Differential virulence of the mite was also hypothesized to explain the survival of VSB. After the first years of varroa invasion in France, most of the untreated colonies were found dead with many mites trapped in entire frames of dead sealed brood. Individual fitness of a mite in those cells was therefore nil. A less virulent parasite, which would not kill the host, would thus have an increased individual fitness. The hypothesis of sub-populations of mites with different levels of virulence was tested using mitochondrial and nuclear microsatellite markers [54,55,56]. The structure of the varroa population in Europe was found to be that of an invasive clone [55]. Therefore, it is unlikely that sub-populations of less virulent mites could explain VSB, or if they were indeed responsible, virulence would be due to a limited number of genes as is the case for varroa populations that have become resistant to the acaricide tau fluvalinate [1,57]. More recently, Beaurepaire et al. (2019) [58] used another set of microsatellite markers and could show significant changes in the genetic structure of the mite populations in different honey bee populations.

Acute bee paralysis virus (ABPV) and deformed wing virus (DWV) are resident in honey bee colonies and become more harmful when associated with varroa, which can transmit them between adult bees and brood and vice versa [59]. Therefore, survival of VSB could be due to a higher tolerance of the bees to those viruses. This hypothesis was tested, and data have shown that the VSB had less ABPV and CPV (chronic paralysis virus) compared to control bees. However, the VSB did not survive longer compared to control bees when injected with the two viruses (Le Conte, personal communication). This suggests that the VSB have fewer viruses because they have fewer mites to transmit virus in the bee population. Nevertheless, it is reasonable to suggest that honey bee resistance, varroa virulence and virus prevalence are constantly under selection pressure and that natural selection favors a co-evolution that secures the survival of both the host and the parasite. 

The effect of the environment and apicultural methods contributing to the survival of VSB cannot be excluded. Those areas where the experiments were carried out are outside France’s major agricultural zone and are very favorable to the development of honey bee colonies. The colonies were manipulated only if necessary and were not moved or managed, as professional beekeeping would recommend. 

#### 2.2.6. Surviving Honey Bees from Norway 

A managed population of local honey bees which had survived for more than nineteen years without varroa treatment in the Østlandet region of Norway were recently the subject of scientific study [60]. Colonies from the population, which were of mixed (“Buckfast”) origin were monitored for mite population levels and mite reproductive success, and two possible resistance mechanisms, grooming behavior and varroa sensitive hygiene (VSH) were evaluated. Mite infestation levels were found to be significantly lower in the survivor colonies compared to control colonies. The authors concluded, however, that whilst reduced mite reproductive success seemed to be a key factor in survival, neither grooming or VSH appeared to be important to explain the differences in survival. More recent investigations have shown that a shorter postcapping period may also contribute to natural colony survival of this population [61], while it is not the case for cell size [62]. Moreover, recapping behavior has been shown to be an important factor in the survival of this bee population [63].

#### 2.2.7. Surviving Honey Bees from the USA

Since 1978, before the arrival of the varroa mite, Tom Seeley studied a unique honey bee population of feral colonies nesting in trees in the Arnot Forest, south of Ithaca, NY, USA. In 2002, 15 years after the arrival of varroa, he observed the survival of the colonies. Inspection of the colonies showed that the population as a whole remained stable over three years despite mite infestation and a comparison with susceptible control colonies did not show differences in mite infestation growth rate [64].

Loftus et al. (2016) [65] hypothesized that the persistence of the feral colonies could be due in part to their habit of nesting in small cavities, and found that the smaller nest cavities and more frequent swarming of feral colonies contributed to their persistence without mite treatments. These results were confirmed by Seeley (2017), studying the life-history traits of the feral honey bees and of feral colonies in small hives, and comparing them with the life-history traits of the bees before the arrival of the mite [66]. He found that young one-year-old colonies survived less well compared to already established colonies. Moreover, established colonies had a mean lifespan of 5–6 years and a queen turnover (swarming) each summer. Using a population model, he demonstrated that these life-history traits can produce a stable population of colonies. Interestingly, the feral colonies in the 1970s and the 2010s have essentially identical sets of life-history traits before and after the arrival of varroa, which suggests that the feral colonies possess defenses against the mite that are not costly. Because feral colonies in the 2010s have to invest in defenses against the mite, Seeley suggests that small colony size and frequent swarming endow them with good defenses against varroa, so they did not have to evolve costly new defenses against the mites. However, he does not exclude the possibility that the feral colonies have needed to evolve some new defenses against *V. destructor*, including hygienic behavior and grooming behavior, but that these new defenses should not be costly. 

#### 2.2.8. Surviving Honey Bees from Russia 

The general rule that in time parasites become less virulent and that their hosts become more resistant, led Tom Rinderer and colleagues at the USDA lab at Baton Rouge, LA, USA, to examine bees from the far east of Russia, where varroa had first been reported to be a problem in the 1950s. Preliminary field studies in the early 1990s led to importations of bees to the USA from the Primorsky region, near Vladivostok in 1997 [67]. After evaluation, these bees were released to commercial breeders in 2000, and studies have shown [68] that the commercially available stocks are indeed more varroa resistant than other commercial strains, and that careful crossing has avoided inbreeding, given the limited original gene pool [69]. Despite twenty years of work, however, the precise mechanisms for the varroa survival of the honey bee colonies remain somewhat unclear, as does the degree to which these bees will survive without varroa treatment. However, it is clear that a number of factors are involved, in particular a reduced number of viable female offspring [70], an increased hygienic response, the removal of infested brood preventing successful mite reproduction, and the removal of phoretic mites through grooming [34,71].

#### 2.2.9. The Case of Wild Honey Bees in Europe 

Wild bees are an important issue within the framework of varroa resistance of honey bees. Whilst colonies kept by beekeepers have a limited chance to evolve varroa resistance because they are systematically treated against the mite, this is not the case for wild colonies, which can be a reservoir for naturally selecting varroa resistance genes. 

In Europe, the spread of varroa and viruses led to the belief that wild colonies had disappeared. Recently, however, Kohl and Rutschmann (2018) [72] made a first assessment of the occurrence and density of wild colonies in natural beech (*Fagus sylvatica* L.) forests in two German woodland areas. It remains unclear, however, whether these colonies indeed constitute a sustainable varroa resistant wild population, or whether they represent recent swarms escaped from nearby surrounding managed apiaries. Based on their findings, they extrapolated that there could exist several thousand wild honey bee colonies in German woodlands. Indeed, the role of forests as a reservoir for the occurrence of sustainable naturally varroa resistant colonies should be taken into account when assessing their role in providing ecoservices to the surrounding area. It has been demonstrated in the USA that feral colonies have lower varroa population growth compared to managed colonies [73]. It would be interesting to know whether wild colonies are similarly spread in natural forests at the European level.

### 2.3. Artificial Selection

One possibility is mass selection. The principle is simple: put together as many varroa infested honey bee colonies as possible in the same place and environment and study their survival when allowed to develop without any treatment for mites, in order to select for varroa resistance. The next year and the next generations, the selection is made on the best surviving colonies. This approach was called the "Bond test" (“Live and let die!”), and has been used successfully in France [74], Sweden [75] and in The Netherlands [76]. 

#### 2.3.1. Gotland Bees 

One example of a varroa-resistant honey bee population resulting from a “Bond test” resides on a small peninsula on the Swedish island of Gotland, where it has been surviving since 1999 without treatment. For the original “Bond test”, 150 colonies were established by the Swedish researcher Ingemar Fries and his colleagues, to study survival rates of untreated colonies and the development of the parasite population under Scandinavian climatic conditions [75]. Shortly after set up, the colonies were provided with an artificial mite infestation. No varroa treatments were performed, but the colonies were inspected and samples were taken in regular intervals. Apart from that, colony management was reduced to a minimum, and the colonies were allowed to swarm freely. Swarms were collected and set up in colonies in the experimental apiary. After three years, the annual colony mortality rate had increased to 80%, after which time it decreased and reached significantly lower levels below 20% after six years [75]. In a similar way, the autumn mite infestation rates of adult bees at first increased dramatically, but then decreased after four years of non-treatment.

Whilst the frequent swarming of the colonies was not found to have a significant effect on the buildup of detrimental mite levels [77], the resistant colonies appeared to have developed adaptive characteristics that allowed them to limit mite population growth, such as a significantly smaller broodnest than non-resistant colonies that were regularly treated [78]. In addition, in a comparative analysis of brood samples, infertile mites and mites with dead offspring were observed significantly more frequently in resistant colonies compared to control colonies [79]. Mites in the Gotland population also showed signs of delayed egg-laying, which has been suggested to result from potential inhibition of egg-laying, maybe through pupal volatiles [52]. In addition, a recent study suggests that virus tolerance, rather than reduced susceptibility or virus resistance, is an important component of the natural survival of the Gotland bees [58,80]. 

#### 2.3.2. Kefuss Bees 

John Kefuss and colleagues [74] initiated their first Bond test in 1993 on 12 *A. m. intermissa* colonies known to be resistant to varroa in Tunisia [81]. These bees were imported from Tunisia to France, near Toulouse. The resistance of these bees was compared with 12 *A. m. carnica* varroa susceptible colonies after exposure to heavy varroa infestations. Only the *A. m. intermissa* colonies survived. These bees hybridized with the local bee populations, and most of the hybrids survived mite infestation, indicating a genetic component of the resistance. 

In 1999, a survival field test was conducted on 268 original European honey bee colonies. After losses of over two-thirds of the colonies, new colonies were made from the survivors. In 2002, genetic material from these survivors was bred into an independent group of 60 colonies. In 2013, 519 non-treated colonies from both groups were being used for commercial beekeeping, and mite populations were very low.

Since 1999, no treatments against varroa have been used by Kefuss et al. in their professional beekeeping enterprise [82]. From this naturally surviving stock, they subsequently select their breeder colonies for economic traits. The best colonies are then tested for hygienic behavior (using a freeze-killed brood assay) and for varroa infestation. Apart from one year, their colony losses are comparable to other beekeepers in the region who still treat their hives with acaricides. The adult bee infestation usually remains below 5% and, according to their report, does not economically justify the use of chemicals. The underlying mechanisms are unknown, but a recent study identified an ecdysone-induced gene significantly linked to resistance; ecdysone initiates metamorphosis in bees and reproduction in varroa [83]. This indicates that under commercial beekeeping conditions, simple methods can be used to select for reduced mite populations. 

#### 2.3.3. Blacquière Bees 

Comparable to the population in Gotland, Blacquière et al. [76] started selecting for surviving colonies in 2007 and 2008, in two isolated locations in The Netherlands. The population of Tiengemeten partly descends maternally from the Gotland (Sweden) population [75]. The population of Amsterdamse Waterleidingduinen is a population of “hybrid” Dutch colonies, established with 70 colonies in 2008, of which 20 were used as controls and 50 as the starting group to select for resistance. No varroa control has been performed since 2007 in Tiengemeten and since 2008 in Amsterdamse Waterleidingduinen [84]. 

The main traits of selection utilized in their approach were the ability of the colonies to grow rapidly (colony growth rate has been determined as a significant predictor of colony success), to survive winter despite the presence of varroa, and then to again develop well in spring. Thus, only those colonies were kept and allowed to produce the following generation that survived the winter, increased in size and produced drones in spring [84]. The different groups of colonies were kept in remote areas during mating (on the island Tiengemeten, or on the Amsterdamse Waterleidingduinen in Lelystad). After significant losses during the first few years, the size of the untreated populations became stable and the colonies now have consistently low levels of mite infestation, varying between 5% and 13% of phoretic mites in broodless conditions [84]. The mechanisms behind mite resistance in these populations are still unclear. The grooming and VSH behavior of these surviving colonies and non-selected control bees were studied. Kruitwagen et al. (2017) [84] investigated grooming behavior at individual, group and colony level, but they did not find differences between the two selected populations and the control population. Panziera et al. (2017) [85] studied the VSH behavior and found that VSH had increased strongly in one of the selected populations, where up to 40% of the infested cells with mites and pupae were removed. However, it had decreased in the Tiengemeten population, compared to the control colonies. The different VSH responses between the two selected resistant honey bee populations lead to the conclusion that more than one mechanism of resistance may have evolved in response to the selection pressure by varroa mites.

After 10 years of such successful program, Blacquière et al. published their selection scheme that they have called “Darwinian black box” selection for resistance [76], so that it can be used by other scientists or beekeepers and promoted the use of honey bees’ natural resilience in beekeeping [86].

### 2.4. Genetic Selection on Chosen Characters

The development of genetic resistance can also result from the successful implementation of deliberate breeding programs that use suitable resistance characters in the selection process of honey bees. The selection for varroa resistance in treated populations has to rely on indirect selection characters, because the direct trait of survivability cannot be studied while the colonies are influenced by veterinary treatments. Starting about 30 years ago, much research in European institutes focused on the identification of suitable selection characters [2,87]. It was based on comparative studies with varroa surviving or resistant colonies to understand the mechanism of varroa resistance in honey bees. In addition to the biological relevance, the heritability and the practicability of testing under field conditions were considered to be of major importance in the implementation of such characters in breeding programs.

In the following paragraphs, we summarize the research on characters that have been used in breeding programs for increased mite resistance. An overview of all characters described here, together with other parameters known to influence mite population development in a honey bee colony is given in Table 1. 

**Figure 1:**
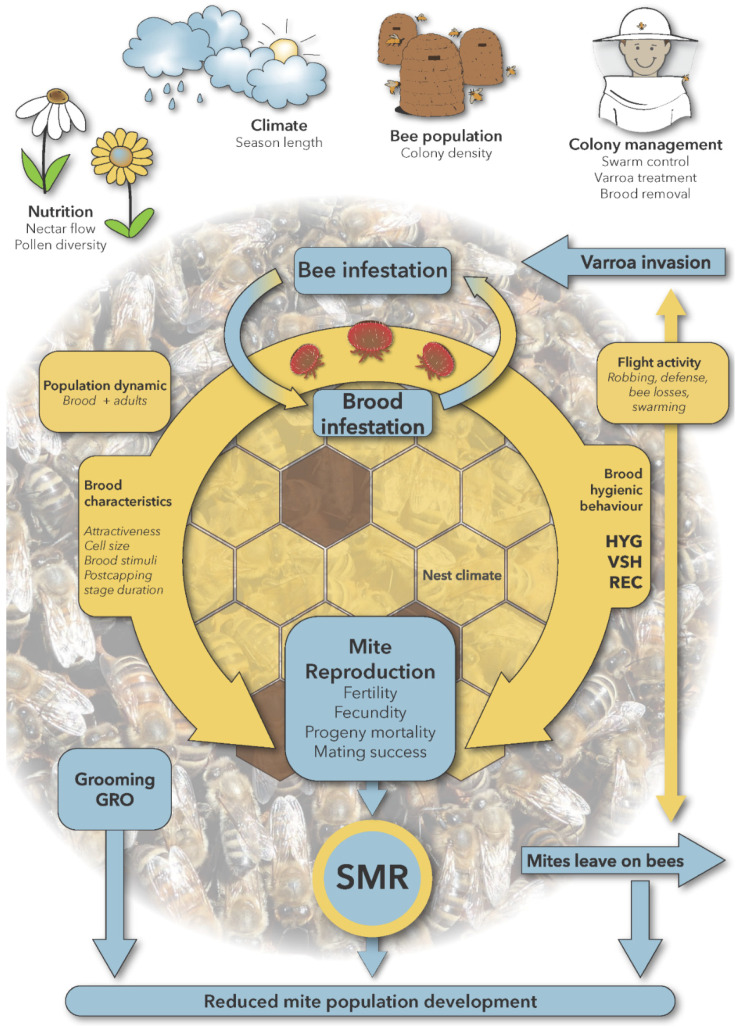
Interactions of mite resistance traits with colony and environmental parameters. Parameters related to the life cycle of the mite are shown in blue (excepted grooming behavior), and characters of the honey bee colony are shown in yellow.

The interactions of resistance traits with colony and environmental parameters are illustrated in Figure 1. 

#### 2.4.1. Hygienic Behavior 

“Hygienic behavior” is the act by which worker honey bees detect and remove diseased or infested brood. The first detailed observations of hygienic behavior were made by American bee scientists in the 1930s, during efforts to determine whether honey bee colonies could in any way be resistant to the bacterial disease American foulbrood (AFB) [95].

Different methods have been developed to test brood hygiene behavior under standardized conditions, but the most common are the freeze-killed [96,97] and pin-killed brood tests [98]. The freeze-killed brood assay is recommended as a more conservative test [99] but it shows higher variability between colonies [100]. The pin-killed brood assay is preferred in most European breeding programs based on its higher repeatability and its correlation with a removal of mite infested brood [101], and its lower cost [100]. A statistical tool has been established to include pin test data in the estimation of breeding values for varroa resistance. Instructions and details on for performing these tests can be found in the COLOSS *BEEBOOK* chapter on standard methods for rearing and selection of *Apis mellifera* queens [94]) and in Facchini et al., 2019 [102], but there is still controversy regarding the reliability and discriminatory capability of the method [100].

The first observations that hygienic behavior is effective in limiting varroa infestation were made by Peng et al., 1987 [18] on *A. cerana*, in an effort to understand the behavioral defense mechanisms in a balanced host-parasite relationship. Subsequent studies showed that the same defense mechanism exists in *A. mellifera* (reviewed by [20]). Hygienic worker bees can un-cap and remove mite-infested cells 4–7 days after the cell is capped [103,104], when offspring of the invading foundress mite are developing on the capped pupa. Hygienic behavior towards the varroa mite has been extensively described by Harbo et al. [93] (see below Varroa Sensitive Hygiene (VSH)) and should not be confused with the hygienic behavior towards larvae infested with AFB or dead brood in general.

Hygienic honey bees have superior olfactory sensitivity compared to non-hygienic honey bees [105,106], which probably depends also on differences in antennal gene expression. Different odorant binding proteins that are significantly correlated with colony hygienic scores have been identified [5,107,108]. 

The removal of infested pupae may theoretically limit the growth of the mite population in three ways: (i) immature mites which have begun to develop in brood cells are killed, decreasing the average number of mated offspring per mother mite; (ii) during the removal process, the mother mites may be damaged or affected with regard to the further reproductive success; (iii) the phoretic period (time spent on an adult bee) of a mother mite is extended if she escapes during the removal process. 

It has been shown that selective breeding can increase the proportion of colonies displaying hygienic behavior and its intensity, and that selection for this trait can be an effective measure to improve resistance of *A. mellifera* against varroa, AFB, EFB and chalkbrood [2,3,109,110,111,112].

#### 2.4.2. Suppressed Mite Reproduction (SMR) 

Much bee breeding work for varroa resistance has taken place at the USDA laboratory at Baton Rouge, LA, USA, by Tom Rinderer, Jeff Harris, John Harbo and colleagues. The primary aim of the program was to selectively breed existing strains of bees widely available in the USA to develop varroa resistant bees that would be suitable for commercial use. Early work focused on measuring mite population growth rate [113,114]. Of four mechanisms of mite resistance that were evaluated in the program (postcapping period (PSD), freeze-killed brood removal, grooming and the non-reproduction of mites) only non-reproduction, which they named “Suppressed Mite Reproduction” (SMR) was found to be correlated with mite population development [113,115,116]. They found that non-reproduction was caused by two heritable traits [117]. 

#### 2.4.3. Varroa Sensitive Hygiene (VSH) 

In 2005, the USDA group at Baton Rouge, LA, USA concluded that the infertility of the mites that they were observing in their breeding trials was linked to the hygienic removal of pupae infested with mites [93,118,119]. As a result, they renamed their breeding program “Varroa Sensitive Hygiene” (VSH) [120]. They developed a bioassay for VSH, which involves inserting combs of mite-infested brood into colonies [92,120,121,122]. Extensive testing of the VSH bees has since taken place under US commercial beekeeping conditions [122,123,124]. In some US breeding programs, resistance levels are reported on website to be high (“100% resistant colonies”).

The European Arista Bee Research foundation organizes, participates and cooperates with beekeepers, institutes and universities in varroa resistance breeding programs. For the ARISTA breeding program, honey bee populations with favorable traits (gentleness, low swarming, and good honey production) are used that are adapted to local climates in order to keep genetic diversity and avoid inbred effects. The ARISTA Varroa Resistance Breeding and Selection Program focuses on the breeding and selection for VSH, improved methodology of varroa infestation measurements, identification of genetic markers and adaptation and improvement of beekeeping practices. For breeding, small colonies with single drone inseminated queens are used. Once established, these colonies are artificially infested with varroa mites. Subsequently, the brood and bee infestation level, SMR and reproduction of the mites are determined. These values are used to estimate the level of resistance. The best colonies are selected for breeding and provided with either single drone or multi-drone inseminated queens in order to maintain the resistance traits as well as to preserve the genetic diversity of the selected populations. To date, there has been no scientific publication, and beekeepers are waiting for stock availability on the market.

#### 2.4.4. Uncapping-Recapping of Varroa Infested Brood Cells 

Social immunity targeting varroa infested brood cells includes removal of mite-infested brood with a notable bias for targeting cells with reproducing mites, a behavior defined within varroa sensitive hygiene (VSH) [119]. This behavior is costly for the colony, because the bee pupa is destroyed by the workers. A less-costly mechanism is the uncapping-recapping behavior, which is more likely to be favored by natural selection, as it reduces mortality of the bee pupae and increases colony competitiveness. Such uncapping of sealed brood cells without removal of the pupae, followed by their recapping, is common in all honey bee populations [46,119,125,126,127,128] and is of low cost for the colony since no brood is sacrificed in the action. Association between recapping and reduced mite reproductive success has been reported from a population bred for VSH [129] and also from four naturally *V. destructor*-surviving populations in Europe [61,63,128], in Brazil and South Africa, [127] and in *A. m. scutellata* as well [46].

Preliminary studies show that mites may leave or invade brood cells while they are temporarily uncapped which can disrupt the fine synchronization of brood and mite development and result in unsuccessful reproduction of mites due to infertility, delayed offspring or missing males (Büchler, unpublished data). However, there may be further negative effects of temporary uncapping on mites inside the brood due to changes of temperature, humidity, kairomone levels or behavioral changes, which need to be investigated.

Investigation of different *A. m. carnica* breeding lines from Austria, Croatia and Germany shows significant genetic differences in the frequency and the targeting of recapping of infested brood cells and indicate a reasonable heritability of this trait [130,131]). This behavior is thus of interest as a character to be used in varroa selection programs. 

It is suggested that VSH, this cost-effective social immunity mechanism, could have evolved rapidly and independently in varroa-surviving or resistant *A. mellifera* populations without sacrificing nestmates, which would provide evidence that honey bees can overcome exotic parasites with simple qualitative and quantitative adaptive shifts in behavior. 

#### 2.4.5. Mite Non-Reproduction (MNR)

Recently, it has been proposed to add a complementary phenotypic definition, mite non-reproduction (MNR), which is the sum of the effects of VSH, Recapping, and SMR induced by immature bees [5,132]. This definition may be important to use in the future to precisely describe which phenotype we are selecting.

#### 2.4.6. Grooming Behavior 

Grooming (GRO) behavior refers to an act that honey bees perform in physically dislodging mites from their bodies by using their mouthparts or legs. Adult bees can remove mites from their own bodies (auto-grooming) or they can be helped by their nestmates (allo-grooming) [18]. A worker bee infested with mites performs a specific dance (grooming dance) to signal the problem. Grooming may injure or kill varroa mites [133], or it may cause mites to either move to other parts of the autogroomer’s body, transfer to a new host or be removed from the bee’s body without causing visible injury [134]. Grooming is a resistance mechanism against *Varroa* spp. in *A. cerana* (reviewed by [11,20]) and has also been observed in *A. mellifera* [104,135], though it does not appear to be as effective as in *A. cerana*. It has been described as an important component to explain the varroa resistance of the gentle Africanized bees in Puerto Rico Island and also in Brazil and Mexico [31,32,33,34,136].

Measurement of grooming behavior in field studies is based on the proportion of mites that drop to hive floors that are damaged, apparently from bees’ mandibles [20,31,137,138]. However, living apparently uninjured mites have been detected in high numbers on bottom board traps [137]. They may also indicate grooming and actually be injured or debilitated [104]. Alternately, they may be healthy, fallen owing to hot weather [138]. In addition, injuries to mites may result from other occurrences as well as from grooming, such as removal of dead mites [139,140] and predation by wax moth larvae and ants [141]. Davis (2009) [142] showed that indentation of the mites’ idiosoma is not damage caused by bees but is acquired during mite development. However, pieces missing from the idiosoma and missing legs cannot be attributed to normal mite development. Thus, careful observation of the fallen mites is required to reliably attribute the presence of the damaged mites to grooming behavior. Whilst a recent study on Carniolan bees from Croatia found no significant correlation between grooming behavior and varroa colony infestation rate [143], another study demonstrated a link between varroa resistance of *A. m. intermissa* colonies and grooming behavior in Algeria [144], which demonstrated that this character can be differently spread between honey bee populations.

After several generations of selection in a test population, colonies selected for this trait have shown significantly more damaged mites and lower infestation rates compared to unselected colonies [145]. However, the estimated heritability was too low (h2 < 0.15) to justify the laborious sample collection and processing in a large-scale selection program [146]). However, other studies have found a very high heritability for this trait [32] as well as a high response to selection [90] and there is evidence of genes affecting this behavior [147,148].

Laboratory assays of grooming using either individual bees or cages of bees have been developed, and produce promising results that correlate with the proportion of damaged mites in source colonies, thus circumventing the difficulty of evaluating damaged mites [31,90,149,150].

#### 2.4.7. Attractiveness of the Brood 

Varroa mites predominantly rely on olfactory triggers to identify and enter brood cells suitable for reproduction. A number of substances produced by the larvae, in particular fatty acids that were already known as brood pheromones, and cuticular hydrocarbons, have been identified as critical substances to attract mites to brood cells shortly before capping [151,152,153]. 

In the context of higher mite resistance of Africanized bees compared to bees of European origin in Mexico, the brood attractiveness of both kinds of bee was compared, and it was found that the brood cells of European-derived bees were parasitized to a much higher degree than the brood cells of Africanized bees [29,30]. Similar results were obtained from Africanized bees in Brazil [154,155], but in laboratory experiments it could also be shown that the components present in the larvae themselves did not differ in their attractiveness towards the mites [155].

#### 2.4.8. Mite Population Dynamics 

In order to select colonies for varroa resistance, regular monitoring of mite populations in order to calculate mite population development is vital. Some breeding programs have been based on simply selecting those colonies with the lowest mite population development without understanding the underlying mechanisms. A number of different sampling methods have been proposed and used to calculate mite populations and population development [2,7,94,156,157], but a key factor for practical use in large scale breeding programs is that the methods need to be simple, reliable and standardized. 

#### 2.4.9. Postcapping Stage 

The postcapping stage duration (PSD) can be calculated as the time between the capping of a cell containing a last stage bee larva by the nurse workers and the time of the emergence of an adult bee from the cell. The female mite enters the cell before capping to reproduce. The first egg that she lays is a male, and then she lays female eggs every 30 h afterwards. In worker brood, a mother mite can usually produce two mature daughter mites before the bee emerges. Female mites need to be mature and mated before the emergence of the young bee from the cell. When there is time for the first daughter mite to mature, the second mite can mature if the postcapping stage duration is around 12 days. 

Moritz and Hänel (1984) [158] demonstrated that in the Cape honey bee, *A. m. capensis*, the reproduction rate of varroa was significantly lower than in *A. m. carnica*. The postcapping stage duration in worker bee cells of *A. m. carnica* and *A. m. capensis* lasts 9.6 ± 0.07 days and 12.04 ± 0.03 days, respectively, which limits the reproduction of varroa in sealed brood cells of *A. m. capensis*. 

There is a medium heritability, but low variability of the average PSD among European subspecies [159,160,161]. However, selection for faster development of worker brood could be quite effective if realized by direct selection on the reproductive individuals (queens and drones) [160,162,163]. 

Wilde and Koeniger (1992) [164] selected a line of bees with a significantly shorter PSD compared to *A. m. carnica* and *A. m. caucasica* control colonies (276.4 h vs. 287.4 and 289.1 h, respectively), but the observed effects on the reproductive success and the population increase in varroa mites in test colonies were not significant [165]. This selection program was stopped after seven generations, recognizing that the achieved breeding progress of 0.2–4.2 h per backcrossed generation remained insignificant [166]. Moreover, selecting honey bees for this trait could induce an arms race for the mite to shorten its own development time. Finally, even if this character has potential to be selected, it is so time consuming that it could not be applied in a beekeeping selection program. Only genomic or proteomic markers could be use efficiently in this context. It does appear, however, that this character can be shown to act as a mite-surviving colony phenotype in naturally resistant colonies in Norway [61].

#### 2.4.10. Brood Cell Size 

As noted above, not all honey bees are exactly the same size, *A. m. scutellata* being slightly smaller than European races, and the question of whether the slightly different size of brood cells that they produce affects susceptibility to varroa has been the subject of scientific investigation [26,49,62]. More recently, there has been much debate among beekeepers themselves based on the idea that the artificially made wax foundation that most beekeepers use has brood cells slightly larger than that of natural comb, and that this has led over a period of time to bees kept in such hives being slightly larger than they would be “naturally”, thus making them more susceptible to varroa. This has led to a movement of “small cell size” beekeepers [167,168], and several manufacturers now offer small (4.9 mm) cell sized foundation. There remains little scientific basis for this movement, and Saucy (2014) [169] has pointed out that much of the debate has been based on a misunderstanding of different historic ways of measuring the exact dimensions of cells. 

#### 2.4.11. Varroa Versus Virus Selection? 

Varroa mites are known to be closely associated with other pathogens, especially viruses [170]. Of particular importance for the understanding of varroa-related colony damage and losses is deformed wing virus (DWV), which has been known as a honey bee pathogen since the 1980s, but in the absence of mites rarely causes overt disease with visible symptoms [171,172]. However, there is now common agreement among scientists that the mites act both as mechanical and as biological vector for DWV. That is, the mites are not only able to transfer virus particles to the bee’s (or pupa’s) hemolymph, but they also play a crucial role in changes in the virulence of this virus.

Recent research has shown that a multitude of different genetic variants of DWV exist, which can be grouped into three main types, A, B and C [173,174,175], which can, however, recombine with each other [176].

Whilst in varroa-free environments (i.e., regions where the mite has not yet arrived) typically a wide range of virus variants exist at low levels, the picture changes dramatically after arrival of the mite. Once the mite is established in an area, the range of virus variants decreases, and at the same time, the numbers of virus copies in bees in infested colonies reach several millions or billions [173,177]. It has also been hypothesized that, as the virus is being passed continually from bee to mite and vice versa, the most virulent virus strains are selected for propagation [173]. Various hypotheses also exist concerning the virulence of certain virus types, and the conditions under which a certain strain achieves dominance over the others [174,178].

It has been shown that varroa-resistant honey bee populations often do harbor significant levels of DWV [40,178,179]), and it has been postulated that part of the varroa resistance might be explained by resistance to the virus [179]. Another hypothesis was put forward by Mordecai et al. (2016b) [178], who suggested that DWV type B might be comparatively benign and could predominantly prevail in varroa-resistant populations. However, a recent study [180] found that all three types of DWV have the potential to cause wing deformities. Our understanding of the complex interactions in the system bee, mite and virus is therefore far from complete, and further research is needed to elucidate the mechanisms and interactions in this system.

#### 2.4.12. Genomic Analysis of Varroa Resistant Bees

One elegant way to search for genetic markers is to use the opportunity of the haploid genome of the drones to link a phenotype to one set of chromosomes and clear the epistasis interactions from genomic analysis [5]. A few studies have used drones from varroa resistant or surviving colonies to successfully identify genetic markers specific to the resistance, such as selective sweeps or QTL [181,182], or, for instance, epistatic interactions [183] on Gotland bees.

Since the publication of the honey bee genome sequence, different teams have tried to identify genes involved in varroa resistance using transcriptomic or proteomic analysis, and most frequently, genes related to olfaction, like odorant binding proteins were identified [5].

Based on the longer postcapping stage duration of drones compared to worker bees, Broeckx et al. [184] focused their study on drone brood from a resilient population from the Amsterdam Water Dunes, The Netherlands. Drones of resistant versus nonresistant colonies were phenotyped on SMR and genetic variants were searched using whole exome sequencing, resulting in eight variants (eight SNPs) in seven different genes that were found to be associated with the drone brood resilient phenotype. Regarding the biological involvement of the identified genes, the authors strongly suggest that this resistance could be linked to better pheromone sensing of adult worker bees as well as a decreases in pheromone release of the larvae which is associated with varroa oogenesis.

Recent scientific programs on this topic are presented in Text S2. Jones et al. [185] have recently published a single nucleotide polymorphism array for selection and breeding on different traits including varroa resistance.

### 2.5. Conclusions from the Literature 

Since the emergence of varroa as a serious pest of *A. mellifera*, considerable time, effort and finance has been devoted to understanding the mechanisms underlying varroa resistance and to breeding bees resistant to the mite. However, progress has often been slow, and some desirable traits, demonstrable in experimental colonies, show low heritability or, alternatively, show benefits that are too small to render them practicable in breeding programs. Another problem is that bee populations apparently resistant to varroa in one location sometimes cease to remain resistant when moved elsewhere and exposed to different environmental conditions or exposed to different mite populations. Nonetheless, significant progress has been made in organized breeding programs and, alternatively, in identifying “survivor” stocks and in understanding the underlying mechanisms of resistance, that may support a more rapid progress in the future.

One aspect that must be considered is that bee populations apparently resistant to varroa in one location sometimes cease to remain resistant when moved elsewhere and exposed to different environmental conditions or exposed to different mite populations. The Europe-wide COLOSS Genotype and Environment Interactions Experiment was carried out in recent years by an international team of scientists [186]. They drew attention to the better survival of locally adapted strains of bee [187] and to the interactions of genetic origin and environment on the occurrence of pests and diseases, especially varroa incidence, in genetically diverse bee populations [186,188,189].

While numerous efforts have been made to describe the mechanisms of the naturally and the artificially selected colonies for varroa resistance, little is known on their extent in honey bee populations and their interest for beekeepers. In the next chapter, we describe the results of the survey we have done on those topics.

## 3. Survey on the Presence of Naturally Selected Resistant Honey Bee Populations and the State of Selection Programs on Varroa Resistance in Across the EU

In the following section, we present the results of a survey on the presence of naturally selected resistant honey bee populations and the state of selection programs on varroa resistance across the EU and some associated countries. We describe the practical experiences of those searching for varroa resistant bees, whether they are bee research institutes, universities, or beekeepers, including those running large commercial operations, enthusiastic breeding groups, and individuals.

For collecting the data for an overview of the EU market for reproductive material of European honey bees, we designed a questionnaire and circulated it among contact persons for each country from our scientific networks: COLOSS (www.coloss.org); the Research Network for Sustainable Bee Breeding (www.beebreeding.net); and SMARTBEES (http://www.smartbees.eu/). This questionnaire (see first line of Table 2) contained questions on the presence of naturally selected honey bee populations and the state of selection programs on varroa resistance in each country. A summary of the 45 answers is presented in Table 2.

The results shows that only seven of the 32 countries acknowledged the presence of naturally selected resistant populations. However, 22 of the 32 countries identified selection programs for varroa resistance, three of which make their products commercially available. Then we conclude that naturally selected populations are not yet largely spread within the honey bee populations, which is not the case for selection programs in the different countries.

After this first step, we went to have further information and directly interviewed experts in the field. Our approach and results are described below.

## 4. Interviews with Experts in the Field (Beekeepers, Breeders, Researchers) to Obtain Information on Practical Experience with Selection for Varroa Resistant Bees

Interviews were carried out with scientists and beekeepers known to be involved in breeding varroa resistant honey bees. We selected these experts based on our own knowledge, and from the relevant networks. We focused on the various varroa resistant honey bee populations, which are known throughout Europe, including both naturally selected populations, and those which have been deliberately genetically selected as part of bee breeding programs. We also included a few international experts from outside Europe.

Data were collected from the returns of the questionnaires (see Text S1) and interviews. As we could not travel or contact everybody for an interview in person, we distributed questionnaires and then if necessary followed up by mail with the different contacts. Forty-eight interviews were completed using the questionnaire from 19 different countries: 41 from Europe; and seven from North America. The questionnaire and the original data are presented as supplementary material (Text S1 and Table S1). Twenty-one breeders are using naturally selected populations, whilst twenty nine genetically select their bees as part of bee breeding programs. Four are using both approaches.

**Figure 2:**
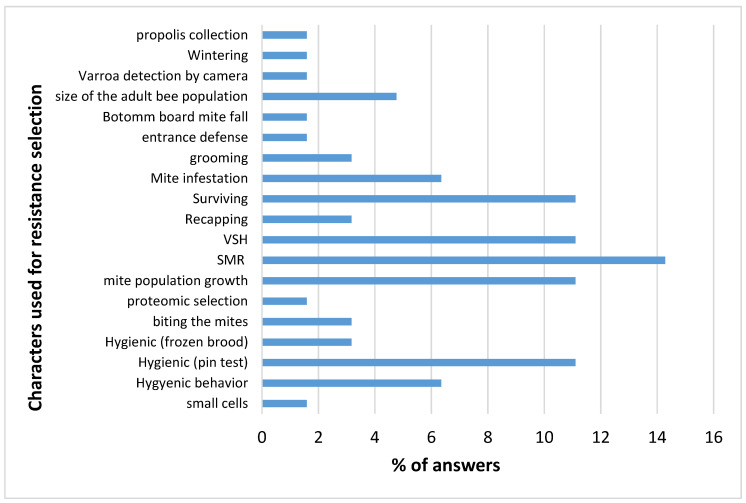
Frequency of different characters used for varroa resistance selection by honey bee breeders. Data are presented as the percentage of breeders using each specific character.

Those breeders using naturally selected bee populations are mostly interested in one main trait: the survival of the colonies. Those breeders selecting their bees for varroa resistance, on the other hand, use 19 different selection characters, with a maximum of five characters per breeder (see Figure 2 and Table S1).

The characters most frequently used are the three linked (SMR, VSH and recapping) characters, mite infestation and population growth, colony survival, and hygienic behavior (including pin test and frozen brood) as reported in Mondet et al., 2020 [5].

The breeders most frequently cited those underlying mechanisms that produce this resistance against mite infestation: surviving of the colonies, VSH, SMR, Pin test and hygienic behavior (Figure 2).

Answers suggested that these mechanisms in different populations were very diverse. The answers focused on the characters that extend within the studied population or between populations. When the breeder used the extent, within the population, for most of their answers, it was notable that SMR and VSH seem to be common worldwide and used by many breeders.

**Figure 3:**
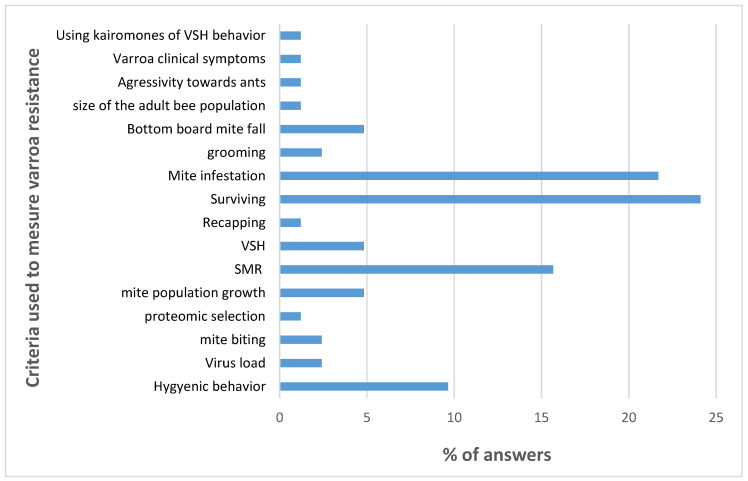
Criteria used for measuring varroa resistance. Data are presented as the percentage of breeders using each specific criterion.

The breeders of naturally surviving populations are using at least one criterion: the survival of their colonies. The breeders deliberately selecting their bees for varroa resistance are using 16 different criteria with a maximum of six criteria per breeder (see Table S1). The criteria most frequently used are mite infestation; VSH/SMR/Recapping; survival of the colony; and hygienic behavior (Figure 3).

The selection strategies are very diverse. Whilst breeders of naturally surviving populations allow their bees to carry out natural selection by themselves, breeders deliberately selecting their bees for varroa resistance usually include one to four characters related to varroa resistance to their already established selection program on, for example, productivity, gentleness, and swarming behavior.

Breeders of naturally surviving populations have generally no mating control, except when they are using an isolated area like an island. Breeders selecting their bees for varroa resistance sometimes have no mating control, but more generally use drone saturated areas for their queen rearing, and/or artificial insemination.

Eleven of the 21 breeders of naturally surviving populations carry out assessment of queen quality, as do 15 of the 28 breeders selecting their bees for varroa resistance. Breeders of naturally surviving populations use colony survival as a trait for selection, so bad quality queens will not survive. Nine of the 21 breeders of naturally surviving populations, and 15 of the 28 breeders selecting their bees for varroa resistance, have local or regional beekeeper collaborations or networks. Most of the collaborations are local or regional. Exceptions are: one group which collaborates with the USDA lab at Baton Rouge (USA) and groups across the EU, one of which acts as a German breeder network of *A. m. carnica* with some active members from neighboring countries, and one group selecting “Buckfast” bees with members mainly from Germany but several other countries as well. In Europe, four breeders of naturally surviving populations were identified by our questionnaires who make their stock available to other beekeepers. Three beekeepers from Greece make all of their stock available One rears queens and sells about 20,000 queens per year at EUR 15 each. Another allows colonies to produce their own queens and rears about 3000 queens per year to sell at EUR 15 to EUR 60 per queen. The third supplies fewer than 300 queens each year for local beekeepers. According to the producers, no varroa treatment is needed. In Norway, less than 500 queens are sold locally for EUR 50 to EUR 70 per queen. This stock is bred and reared as a commercially viable stock for the southern regions of Norway and for some commercial honey production. It is classified as “Buckfast”, and no varroa treatment needed according to the producer. In Puerto Rico, queens are distributed to participating beekeepers from the only breeding center, but no stock is available for sale. According to the producer, no varroa treatment is needed.

Fifteen of the 28 breeders selecting their bees for varroa resistance make their stock available to beekeepers. Most of the breeders include at least one trait for varroa selection in their selection program based on productivity, gentleness and swarming tendency. Only four breeders produce queens that are actually claimed to be varroa resistant without the need of control measures: one in Finland; one in France and two from the USA. In Finland, one beekeeping operation sells varroa resistant queens at EUR 500 per queen. In France, one beekeeper supplies queens at about EUR 10 per virgin queen. In the USA, scientists supply breeding material to queen producers at a price of USD 200 to USD 350 per queen. Whether these queens are actually 100% varroa resistant under all conditions is untested and needs to be confirmed. In Germany, one beekeeper supplies SMR selected queens only to beekeepers seriously interested in varroa resistance breeding and is propagating the stock via drone mother colonies. According to the breeder no varroa treatment is needed. Members of one breeding association offer *A. m. carnica* queens, and those of another one offer “Buckfast” queens, which are supposed to have improved resistance traits, especially for hygienic behavior and SMR, combined with excellent commercial traits. Some of them cooperate, especially in the selection for SMR in well-established breeding lines. There is a high demand for such queens at the national and international level. According to breeders’ experience, such stock can usually be managed with a reduced chemical treatment regime, but not without any kind of treatment.

Only a few positive answers were provided on location and numbers of breeding and training centers. One breeder in France provides training. Five groups provide training within the framework of varroa resistance selection programs. Only four breeding training centers were identified: in Kirchhain/Germany, in Olsztyn/Poland, in Mugla, Turkey, and one in Puerto Rico.

There were very different answers to commercial attractiveness to this topic, depending on the type of selection and the design of the varroa selection program. In all cases, the basic attraction was to reduce colony losses, while avoiding the need to chemically treat the colonies. Breeders of naturally surviving populations tend to sell their queens for use in a similar environment, as this should play a role in the equilibrium between the varroa mites and their host colonies. Besides a lack of selection for commercial traits in some of those populations, this is why the commercial attractiveness for those queens could be limited. However, in Norway, the commercial attractiveness is important as the stock (“Buckfast”) is bred and reared as a commercially viable stock for the southern regions of Norway, which gives the proof of concept.

Breeders selecting their bees for varroa resistance combined with other commercially attractive traits such as production, gentleness and swarming, experience different levels of attention for their breeding products. In general, an increasing demand for queens is observed by many German breeders, but most of them are cautious to advertise their stock with the label “resistant” as there are only a very few examples of highly varroa resistant bees produced. However, in the USA, there is a clear demand, greater than the supply, for such bees.

Breeders of naturally surviving populations are mostly interested in having colonies surviving varroa, so productivity is of secondary importance. This is not, however, the case for breeders selecting their bees for varroa resistance which include varroa resistance among other commercially attractive traits such as production, gentleness and swarming. No breeding values were recorded, with exception of a group of German *carnica* breeders (Arbeitsgemeinschaft Toleranzzucht–AGT), who use the www.beebreed.eu database system.

### 4.1. Naturally Selected Populations

In Greece, five beekeepers use varroa resistant colonies that they select on their survival ability. They seem to be successful, as three of them sell thousands of queens. In Italy, three beekeeping operations have used naturally selected colonies for five to 15 years and plan to develop them in protected areas. In The Netherlands, one operation has maintained a surviving population on Texel Island for 15 years, but the availability of the material is limited. There is also one survival program started in The Netherlands, which seems to be successful, but there will be no stock available for some time. In Norway, one varroa survival population has been maintained since 1998 and seems successful. Queens are sold locally and abroad for approximately EUR 50 per mated queen. This operation produces an average of 400 queens per season from a single geographical breeding center. In France, Yves Le Conte has maintained two populations of naturally resistant bees (see the chapter on this issue) since 1999, but no material is available for sale.

Outside Europe, in Puerto Rico, gentle Africanized bees which are naturally varroa resistant are available for beekeepers. About 1000 queens per year are produced in one breeding and training center. The Arnot Forest bee population identified by Tom Seeley in the USA is not available to beekeepers.

### 4.2. Deliberately Genetically Selected Populations

In France, one beekeeper has for many years sold virgin queens from his “Bond test” bees at a low price of EUR 10 per queen. In Finland one group have not treated their colonies since 2008 and produce queens. In Germany, a beekeeper produces varroa resistant queens only for beekeepers interested in varroa resistance breeding. Several breeders from Germany and neighboring countries offer queens from their lines selected on SMR and further resistance traits, but most of them avoid advertising them as varroa resistant as this is not yet proven under different environmental and management conditions.

In Sweden, the Gotland bee population is not available for beekeepers.

Outside Europe, the USDA Baton Rouge team have been leaders in this field for many years and developed the VSH and SMR methods that are now used by many beekeeping operations and scientists throughout the world. They have conclusively demonstrated that selection using these characters can be efficient.

Prices for resistant breeder queens seem to be very variable, in the range of EUR 10–500.

## 5. Conclusions about Varroa Resistant Honey Bees in Europe

If we focus on the various varroa resistant honey bee populations (that is, those for which there is no need for varroa treatment) in the EU, we identified seven countries which have naturally selected varroa resistant populations (France, Italy, Ireland, Lithuania, The Netherlands, Norway and Sweden), and several where bee breeding programs focusing on varroa resistance are being conducted, albeit often on a small scale. Supplies of queens are, however, very limited in most areas; alternatively, breeders participating in selection programs are often very cautious about advertising their stock as “resistant”. However, several countries have recently initiated new selection and breeding programs, so it is clear that there is an increasing interest in developing these aspects, either by using naturally varroa resistant bees, by adding suitable selection characters to existing selection schemes, or by devising entirely new programs. A recent survey made in Switzerland demonstrated that many beekeepers are interested in developing a breeding strategy for resistant stock even though the bees would produce less honey, swarm more often or be less gentle, showing a clear desirability for resistance traits [190]. There may also be many naturally resistant populations, which have yet to be identified. It is necessary to strengthen cooperation among beekeepers and breeders and to develop sustainable and effective infrastructures for the promotion of varroa resistant and commercially attractive honey bee stocks in the EU.

## Figures and Tables

**Table 1:** Varroa resistance in honey bees—Definition of relevant characters and parameters.

Character/Parameter	Abbr.	Measures	Description and Remarks	References
Bee infestation		Mites/g of bees	Proportion of phoretic mites on adult bees	Rosenkranz et al. 2010 [22]
Mites leave on bees		Bees per day	Loss of bees inside and outside the hive due to mortality and drifting, attached mites are lost	Büchler et al. 2010 [2]
Brood attractiveness			Relative brood infestation of different brood samples under uniform infestation pressure, expressed also as the ratio of mites on bees to mites in brood cells	Rosenkranz et al. 2010 [22]
Brood dynamic			Change over time in the number of worker and drone brood cells of a colony	Rosenkranz et al. 2010 [22]
Brood infestation		(adult) mites per brood cell	Proportion of mites in brood cells	Rosenkranz et al. 2010 [22]
Brood stimuli			Response causing agents, which are produced by the brood	Mondet et al., 2016 [88]
Cell size		Diameter of cells [mm]	smaller cells may affect mite infestation and reproduction	Winston, 1987 [89]
Defense		Scores	Aggressive behavior of worker bees in order to protect the colony	Winston, 1987 [89]
Fecundity		Number of offspring per individual	Potential for reproduction, e.g. max. number of offspring per reproductive cycle	Rosenkranz et al. 2010 [22]
Fertility		Presence of offspring	Capability to produce offspring	Rosenkranz et al. 2010 [22]
Flight activity		Returning bees/min	Flying of worker bees, infested bees or attached mites can be lost in the field	Rosenkranz et al. 2010 [22]
Grooming behavior	GRO	Mite injuries and mite removal	Worker bees detect, remove, damage or destroy the mite from themselves or other workers	Morfin et al., 2020 [90] Guzman-Novoa et al., 2012 [34]
Hygienic behavior	HYG	Freeze-killed or pinkilled brood	General hygiene behavior towards diseased/infested brood cells	Dietemann et al., 2013 [7]
Varroa invasion		Mites/interval	Mites enter a colony, transported by worker bees	Rosenkranz et al. 2010 [22]
Mite mating success			Rate of successfully impregnated female mites	Rosenkranz et al. 2010 [22]
Mite mortality		Dead mites on bottom board	Death rate of mites	Dietemann et al., 2013 [7]
Mite reproduction	MR	see with SMR Rate of reproductive mites from single infested worker brood cells	Production of varroa offspring	Mondet et al., 2020 [5]
Nest climate			Temperature and humidity level and variation in the brood nest, affects postcapping stage duration and mite reproduction	Kraus et al., 1997 [91]
Population dynamic			Change over time in the number and ratio of worker and drone brood and bees in a colony, influenced by season, swarming, etc.	Büchler et al. 2010 [2]
Postcapping stage duration	PCD	Hours between cell capping and emergence	Time span between closing of brood cell and emergence of adult bee. A prolonged postcapping period of the brood cell should reduce the number of mature adult daughter mites. However, a shorter developmental time of the brood probably permits one brood cycle more per season.	Rosenkranz et al. 2010 [22]
Progeny mortality		Dead offspring in cells	Death rate of mite offspring	Villa et al., 2009 [92]
Recapping	REC	Inner side of cell cap without cocoon	Opening and closing (recapping) of sealed brood cells, may be non-infested or infested	Oddie et al. 2018 [63]
Reduced mite population development	MPD	Comparison of infestation levels during time	Attenuated increase in the number of mites per colony; is affected by mite reproduction, population dynamics, grooming and flight activity (varroa invasion, mites that leave on bees)	Büchler et al., 2010 [2]
Robbing			Worker bees steal honey from foreign colonies, phoretic mites may leave or enter a colony	Winston, 1987 [89]
Suppressed mite reproduction	SMR	Rate of non-reproductive mites from single infested worker brood cells	Heritable trait of the honey bee that negatively influences varroa reproduction	Harbo and Harris, 2005 [93]
Swarming		Scores	Reproduction of the colony: the old queen leaves with about half of the worker bees, brood interruption and split of mite population reduces infestation level	Fries et al., 2003 [77], Büchler et al., 2013 [94]
Varroa sensitive hygiene	VSH	Rate of removal from all infested worker brood cells	Uncapping and removal of Varroa infested brood cells	Villa et al., 2009 [92]

**Table 2:** Overview of varroa-resistant populations of honey bees in Europe, both naturally selected and from selection programs, and their commercial availability for beekeepers.

Country	Naturally Selected Resistant Populations Present	If Yes, How Many	Proven Examples of Survivor-Stock	If Yes, How Many	Are There Selection Programs for Varroa Resistance	If Yes, are Queens Commercially Available?	If Yes, at What Price
**Austria**	none known		No		yes, two	Yes, but not marketed as ‘resistant’	no data
**Belgium**	none known		no		yes, starting: three	no	
**Bulgaria**	anecdotal reports		no data available.		no; but selection on hygienic behavior carried out		
**Croatia**	anecdotal reports		no		yes	no	
**Cyprus**	none known		no		no		
**Czech Republic**	none known		no		no, but selection on hygienic behavior		
**Denmark**	none known		no		Just initiated. No sale, no price.		
**Estonia**	none known		no		no		
**Finland**	none known		no		starting		
**France**	yes	3: Avignon, Sarthe, Tarn			yes, several initiatives	no	
**Germany**	none known		anecdotal reports		yes, several initiatives, at least three	Yes, but not marketed as ‘resistant’	EUR 50–EUR 75
**Greece**	anecdotal reports		anecdotal reports		few-only individual initiatives	no	
**Hungary**	no		no		yes, based on VSH	no	
**Ireland**	anecdotal reports		no		yes	no	
**Italy**	yes	4: Liguria (A.m.m.), Eolie (A.m.s.), Vicenza	Gorgona (A.m.l.),		yes (one public and two private breeders (Ligustica, and Carnica-mix).	no	
**Latvia**	none known		no		no		
**Lithuania**	yes	local hybridized bees of A.m mellifera near Belarus border	anecdotal reports		no		
**Luxembourg**							
**Malta**	none known		no		starting (Smartbees project)	no	
**Netherlands**	yes	3: Lelystad, Tiengemeten and at Laren	anecdotal reports		yes, 4 in total	no	
**Poland**	none known		no		yes	no	
**Portugal**	none known		no		no		
**Romania**	none known		no		starting (Smartbees project)	no	
**Slovakia**	none known		no		yes, to some extent	yes	EUR 20
**Slovenia**	none known		no		yes	no	
**Spain**	none known		no		starting (Smartbees project)	no	
**Sweden**	yes	southern Gotland (the Bond bees)	anecdotal reports.		yes, one based on VSH, and some other small projects	no	
**United Kingdom**	anecdotal reports	Lleyn peninsula	possibly…		yes, one University program (hygienic)	sometimes, small scale	no data
**Israel**	none known		no		no		
**Macedonia**	none known		no		no		
**Norway**	yes	one in southeast Norway, untreated since 1997	no		few initiatives (Smartbees)	very limited amounts	EUR 75
**Switzerland**	none known		anecdotal reports		no		
**Turkey**	yes	Marmara Island	no		yes, based on Marmara Island stock	yes	EUR 15–EUR 20

## Supplementary Materials

The following are available online at https://www.mdpi.com/2075-4450/11/12/873/s1, Text S1: Standard questionnaire for the expert interviews; Text S2 Recent scientific programs on Varroa resistant honey bee selection using genetic markers; Table S1: beekeepers and breeders experience.
