# Supplementary material for: Geographical Distribution and Selection of European Honey Bees Resistant to Varroa destructor
Source: Insects. 2020 Dec 8;11(12):873. doi: 10.3390/insects11120873 (PMC7764010; doi:10.3390/insects11120873)
Supplement: Supplementary file 1 [file insects-11-00873-s001.zip › SMZip/Table S1 beekeepers and breeders experience .docx]

**Table S1: beekeepers and breeders experience**

| **Country** | **Naturally selected populations** | **genetically selected populations** | | **mechanisms** | | |
| --- | --- | --- | --- | --- | --- | --- |
|  |  | **Y/N** | **What character?** | **underlying mechanisms** | **extent of underlying mechanisms ?** | **criteria for measuring varroa resistance** |
| **Austria** | N | Y | surviving on small cells, HYG GRO | Small cells  GRO | GRO high in A.m. m; small cells; HYG high in A.m. c | HYG all over the year; GRO in Oct and Nov |
| **Austria** | N | Y | Mite mortality, HYG, SMR | HYG and VSH |  | mite population development, HYG, end of brood activity in autumn |
| **Austria** | N | Y | VKF (“Varroa Killer Factor” = killed mites/ total mites) | 1. Killing of mites, 2. Infertility in brood, 3. Removing infested brood, 4. Brood not attractive for mites | Varroa killer factor of the bred colonies per year increased from 27% in 1990 to 96% in 2008. | grooming: natural fall and mites with injuries result in a Varroa-Killer-Factor |
| **Canada** | N | Y | Using proteomic selection |  | No | survival for 1 y without varroa treatment; proteomic panel. |
| **Canada Ontario** | N | Y | mite population growth | grooming behavior and varroa sensitive hygienic behavior | No |  |
| **Croatia** | N | Y | since 2005 varroa mite population growth and HYG (pin test). SMR and REC selection, Since 2017 survival testing | VSH, but also HYG and REC | Not known | SMR and recapping, Natural mite fall , HYG, lowest varroa infestation or growth, survival test |
| **Croatia** | N | Y | HYG and infestation levels |  |  |  |
| **France** | N | Y | Survival (Bond Test) | HYG | locally | suvival, mite infestation |
| **France** | Y | N | Survival | SMR, VSH, olfaction | Locally | survival |
| **Finland** | N | Y | survival, low mite number | olfaction | No | Sugar shake, survival, general conditions |
| **Germany** | N | Y | survivability, GRO, defense, constant natural mite fall, threshold of 3% mite infestation | local environmental impacts, flow, beekeepers managements, hive microorganisms, traits of queen`s stock , selection by beekeeper | small part | Counting mite drop, sugar shakes, watching virus disease, Survivability in winter |
| **Germany** | N | Y | low mite population increase; HYG (pin test); high SMR and REC | Well adapted brood cycle, VSH and REC behavior (resulting in SMR), GRO | significant differences between different Carnica breeding lines for the selected characters |  |
| **Germany** | Y, offspring from imported Primorski and Monticola | Y | natural mite fall, VSH/SMR, survival when untreated; to some extent GRO | mite fall is regularly measured, VSH/SMR only estimated in some colonies - broad range observed | Large variability, up to 87,5% to 100% VSH (ARISTA method) | Mite infestation VSH/SMR, survival without treatment |
| **Country** | **Naturally selected populations** | **genetically selected populations** | | **mechanisms** | | |
|  |  | **Y/N** | **What character?** | **underlying mechanisms** | **extent of underlying mechanisms ?** | **criteria for measuring Varroa resistance** |
| **Germany** | N | Y | traditional characters (gentleness, swarming, honey) plus HYG | bees must detect infested cells/ damaged larvae | could not find differences between populations | not removing drone brood to force colonies to develop under infestation pressure |
| **Germany** | Y | Y | reduced mite reproduction, survival without treatment | SMR, mites not entering colony | Y | Survival of the colonies |
| **Germany** | N | Y | VSH - ability of individual workers to detect Varroa infested brood cells via infra-red video technique, pin-tests, Varroa population growth and since 3 year survival without Varroa treatment | Ability of workers to detect the degree damage caused by parasites or diseases | No | Hygienic behavior, pin-tests, Varroa population growth, survival without Varroa treatment |
| **Greece** | Y | N |  | maybe resistance to viruses |  | Highly productive colonies that survive without as mother colonies; highly productive colonies from last year with early buildup as drone mothers |
| **Greece** | Y | N |  | build their own combs every year; Not known |  | Select from survivors |
| **Greece** | Y | N |  | REC | no | 1. Build up in the Autumn, after they show DWV symptoms 2. build up of the colony to the 2nd floor during the 2nd year |
| **Greece** | Y | N |  | Hygienic behaviour , probably tolerant to viruses | no | 1. High HYG (freeze brood) 2. productivity above threshold of 35 kg. |
| **Greece** | Y | N |  | Not known | no | Survival |
| **Ireland** | Y | Y | HYG using liquid nitrogen | HYG | The level of HYG varied a lot in subsequent F1 and F2 generations (from 20% to 95%). | Varroa counts |
| **Italy** | N | N | Docility, brood pattern, overwintering ability, spring drone production, summer brood interruption | No | No | Adult bee infestation index, July and October |
| **Italy** | Y | N |  | attention to the varroa; resistance toward DWV and to the isolation condition of the varroa population instead to the honeybee resistance mechanisms | HB population in Pianosa island, highly infested by varroa but very few loss of colonies is recorded | monitoring the DWV titre in both bees and varroa |

| **Country** | **Naturally selected populations** | **genetically selected populations** | | **mechanisms** | | |
| --- | --- | --- | --- | --- | --- | --- |
|  |  | **Y/N** | **What character?** | **underlying mechanisms** | **extent of underlying mechanisms ?** | **criteria for measuring Varroa resistance** |
| **Italy** | N | Y | Hygienic behavior (pin test), Varroa population growth, SMR, gentleness, honey production | Stock not classified as “resistant” yet. Breeding project in 2014; increase in HYG and SMR | No | Varroa infestation levels in different moments, and calculation of breeding values (on [www.beebreed.eu](http://www.beebreed.eu) platform) |
| **Italy** | N | Y | All traits with economic interest for beekeeper, searching for the highest expression of VSH | VSH | ? | Calculate infertility value and total number |
| **Italy** | N | Y | Survival: bond test on Lunden bees fron Finland | grooming, VSH, resistance to viruses, bacterial and fungal diseases | one less treatment necessary – or treatment 2 months later than before | survival test |
| **Italy** | Y | N |  | Preserved environment, Soft varroa treatments, presence of feral colonies | ? | Colony survival |
| **Lithuania** | N | N |  |  |  |  |
| **Luxembourg** | N | Y | 11 criteria used in Buckfast breeding; most importantly vitality and disease resistance of bees and brood; high wintering ability, gentleness, fecundity, honey collecting | VSH and SMR | Y | Number of mites after treatment in production colonies, artificial infection and subsequent brood observation in non-production colonies |
| **Luxembourg** | N | Y |  | brood effect, VSH | 10% of the crossings in the first generation of selection has significant levels of resistance | infestation level, SMR |
| **Netherland** | Y | N |  | the brood nest at 34.5 degrees, SMR | No, observations only on Texel population | Delayed egg laying and non-reproduction |
| **Netherland** | Y | N |  | mite populations in the colonies grow slower, fertility, fecundity, VSH | VSH, but not GRO | measured mite infestation twice per year |
| **Norway** | Y | Y | survival, colony strength, honey production, low swarming drive and some selection for gentleness. | reduced mite reproduction, recapping, shorter developmental time as capped brood. GRO and VSH **does not** seem to play an important role (Oddie et al. 2017). 4.9 mm wax foundation. short season for brood rearing | See Oddie et al, 2017 for details | lack of clinical symptoms of high varroa infestation: few mites on adult bees; aggressiveness towards ants; low mite infestation in drone brood; Very few bees with deformed wings; Normal colony development |

| **Country** | **Naturally selected populations** | **genetically selected populations** | | **mechanisms** | | |
| --- | --- | --- | --- | --- | --- | --- |
|  |  | **Y/N** | **What character?** | **underlying mechanisms** | **extent of underlying mechanisms ?** | **criteria for measuring Varroa resistance** |
| **Norway** | Y | N |  | Could be: VSH, GRO, REC, small colony size, elevated swarming and resistance/tolerance to DWV and recombinants | combination of traits : reducing Varroa reproductive success or becoming resistance to the viral dynamics cause by high-level infestations | The ability of a population to persist without human-mediated mite treatment. Lower numbers of mites on bottom boards. |
| **Poland** | N | Y | Honey production, gentleness, swarming, overwintering, fast development, hygienic behavior, grooming behavior, SMR | HYG, GRO | no | HYG, GRO, SMR |
| **Poland** | N | Y | Honey production, development, swarming behavior, wintering, calmness, defensive behavior, HYG (Pin test), spring infestation with Varroa, seasonal infestation development, SMR | HYG, reproduction of Varroa, VSH, GRO | Yes | Mite infestation (natural mite infestation, infestation of bee samples, infestation of bee brood) ,Pin test 3 times per year, VSH, SMR |
| **Puerto Rico** | Y | N |  | GRO. Genetic markers in the region of the linkage group associated with Varroa grooming genes. | GRO occurs on nearly 100% of the colonies tested. The number of individuals responding with biting in each colony ranges from 40 to 90%. | Varroa mites in worker brood. Adult bees with Varroa (adult bees in ethanol, and on shaker) |
| **Slovenia** | N | Y, but not focused on Varroa | 1) coloration (grey) 2) cubital index (2.4 – 3.2) 3) calmness 4) honey yield 5) low swarming | No | No | pin-test in combination with sugar test |
| **Spain** | Y |  |  | Natural selection | ? | long-run survival of colonies; Levels of infestation |
| **Sweden** | Y |  |  | reduced colony size, reduced mite reproduction, and a tolerance to virus | supposed global | fewer successfully reproducing mites |
| **Sweden** | N | Y | VSH | Genetics | It is present in all tested populations (Buckfast, Ligustica, Carnica and Nordica) but in diluted form | Varroa mite growth test during May-August by washing bee samples; VSH screening by opening brood cells and counting fertile/infertile varroa |

| **Country** | **Naturally selected populations** | **genetically selected populations** | | **mechanisms** | | |
| --- | --- | --- | --- | --- | --- | --- |
|  |  | **Y/N** | **What character?** | **underlying mechanisms** | **extent of underlying mechanisms ?** | **criteria for measuring Varroa resistance** |
| **Turkey** | Yes, Mugla ecotype of A. m. anatoliaca | N |  | many factors affecting resistance | according to the literature | varroa infestation in bees and brood. Natural mite fall. Colony size |
| **UK** | N | Y | HYG | Hygeinic behaviour. Demonstrated to have lower incidence of deformed wing virus. | See Al Toufailia et al, 2014 for details. | Freeze killed brood test, varroa infestation of bees and brood. |
| **USA** | Y |  |  | Killing adults by GRO, and killing brood by VSH/REC | Only one population | Survival without treatment for more than 20 years |
| **USA** | N | Y | VSH | ability of the worker bee to recognize and remove the reproductive mites in brood cells | but in my opinion, the trait is relatively common in honey bees worldwide. | measuring mite reproduction in worker brood cells: see: harbobeeco.com |
| **USA** | N | Y | VSH, size of the adult bee population, honey production | Expression of VSH results in increased removal of mite-infested brood and suppresses mite population growth | Non-reproduction of Varroa typically is >70% in bees selected for VSH and <50% in unselected bees | mite infestation in the late summer or autumn or season-long mite-population growth |
| **USA** | N | Y | winter survival, hygienic behavior, propolis collection, low mite population growth over the winter, gentleness and honey production. | Program starting! hygienic removal of mite-infested brood, removal of virus infested brood, and low mite population growth |  | Freeze-killed brood test, testing compounds that elicit removal by both MN-HYG and VSH lines of bees |

**Table: beekeepers and breeders experience (continued)**

| **Country** | **Selection** | | | | |
| --- | --- | --- | --- | --- | --- |
|  | **selection strategies** | **mating control** | **mating desing** | **assessment of queen quality** | **collaboration networks** |
| **Austria** |  | Instrumental insemination; mating station in the Alps |  | important | N |
| **Austria** |  | y | y | y | y |
| **Austria** | Selection of colonies with highest VKF as breeder colonies next year | y | yes | y | n |
| **Canada** | Working with local bee breeders, we use a combination of the above-described proteomic test and standard N_2_ freeze-killed brood testing | Yes | Closed mating | No | Yes, extensive |
| **Canada Ontario** | Mite population increase (times fold) from spring to summer using sticky papers | Yes | mate the queens on an island in Lake Simcoe | No | Yes |
| **Croatia** |  |  |  | queens are not tested for mite resistance |  |
| **Croatia** |  |  |  |  |  |
| **France** |  | Y | Y | Y | limited |
| **France** | survival and breeding on the best colonies | N | N | N | with local beekeepers |
| **Finland** |  | y | y | n | y |
| **Germany** | use a threshold of 3% to 5% mites in hive, the thriving after winter, survivability, accommodation of brood nest to stores, local adaption | N | N | Y | Y |
| **Germany** | use well-established, gentle and productive breeding stock and try to improve the mentioned resistance characters by performance testing and selective breeding | Y | on isolated islands and artificial insemination | yes, standardized testing protocol and breeding value estimation | yes, cooperation with many private breeders within the breeder association |
| **Country** | **Selection** | | | | |
|  | **selection strategies** | **mating control** | **mating desing** | **assessment of queen quality** | **collaboration networks** |
| **Germany** | selection based on low mite infestation, VSH/SMR, survival without treatment | y | y | y | y |
| **Germany** |  | y | y | y | y |
| **Germany** |  | y | y | y | y |
| **Germany** | Selection of bees with a higher odor sensitivity and find genetic markers for this trait | Y | Y | Y | local or regional collaborations and networks |
| **Greece** | a) productivity in 3 consecutive periods of the year.. b) Survival without treatment c) no swarming the previous year d) calm, not agressive | Saturation with selected drones |  | Not really- only visible cues (quality and quantity of brood) | Some collaboration |
| **Greece** | a) productivity in honey; b) quick colony buildup c) Survival without treatment | Saturation of drones |  | building up in the season | no |
| **Greece** | a) no stimulation in spring; b) examine for DWV in autumn , select the ones they have less; c) stimulate brood production after that d) select the ones they build up a lot in the 2nd year | no |  | low build up, no swarming, no diseases, much pollen, productivity | no |
| **Greece** | a) producing at least 35 kg of honey b) HYG test freeze killed brood c) not swarming and calmness | many drones from the rest of the colonies |  | brood pattern, swarming tendency, HYG | no |
| **Greece** | a) survival of the colonies with most varroa (+ brood from the honey production colonies); b) not much manipulation; c) not intensive apiculture managments | nothing |  | a) calmness  b) productivity | no |

| **Country** | **Selection** | | | | |
| --- | --- | --- | --- | --- | --- |
|  | **selection strategies** | **mating control** | **mating desing** | **assessment of queen quality** | **collaboration networks** |
| **Ireland** | HYG - colonies with > 90% removal of frozen brood will be selected; focused on Varroa numbers, evidence of uncapping and Varroa mite reproduction. | area will be saturated with drones from selected colonies, and insemination |  | ? | Beekeepers |
| **Italy** | no | no | no | no | no |
| **Italy** | no | no | no | no | no |
| **Italy** | Selected stock used for drone production in protected mating station; virgin queens from local beekeepers participating in project can be mated here | Yes | Yes | No | Yes |
| **Italy** |  | Yes | Yes | No | No |
| **Italy** | varroa resistance + commercial traits (mainly productivity) | No strict control but strong production of drones | n | n | n |
| **Italy** | reproduce the best colonies, split them | n | n | n | n |
| **Lithuania** |  |  |  |  |  |
| **Luxembourg** |  | Y, artificial insemination | Y | Y | Y Buckfast breeders |
| **Luxembourg** | starting with the best productive bees, single drone insemination (in combination with multi drone mating) | Y | Y | Y | both regionally as well as more broadly (EU-wide) |
| **Netherland** | non-intervention | on Texel Island | No | No | provide queens and mating possibilities |
| **Netherland** | Survival, Fitness (includes timely reproduction) | N | Y, remote isolated mating area, within population mating | N | N |

| **Country** | **Selection** | | | | |
| --- | --- | --- | --- | --- | --- |
|  | **selection strategies** | **mating control** | **mating desing** | **assessment of queen quality** | **collaboration networks** |
| **Norway** |  | no strict mating design or mating control. | Queens in the badly performing colonies are changed ASAP whereas queens in average performing colonies are changed every second year |  | cooperation with another commercial beekeeper using more or less the same stock of bees. |
| **Norway** | hygienic, using small black ants and the bees’ reactions to them at the entrance and within the colony. | no | no | no | no |
| **Poland** | next generation the colonies with the highest selection index | yes |  | yes |  |
| **Poland** | ranking by total points assessment of the characteristics with particular emphasis on the survival of HB and SMR | Y | instrumental insemination | Y | N |
| **Puerto Rico** | no selection at this date | N | Y | Y | Y |
| **Slovenia** | none | No | No | Yes | No |
| **Spain** |  |  |  | Y |  |
| **Sweden** | natural |  | randomly selecting mother colonies and natural mating within the isolated population | phenotype colonies for mite reproductive success | Gotland bees are not part of any breeding networks |
| **Sweden** | Insemination | ? | ? | ? | ? |
| **Turkey** | In 2015, start with 100 colonies. index methods apply in order to select queen bees and drones. | Y | Y | N | N |

| **Country** | **Selection** | | | | |
| --- | --- | --- | --- | --- | --- |
|  | **selection strategies** | **mating control** | **mating desing** | **assessment of queen quality** | **collaboration networks** |
| **UK** | Select for highest scores on freeze killed brood test | Open mating and sometimes instrumental insemination |  | Some tested queens sometimes available | Beekeeping associations |
| **USA** | Reliance on natural selection | N | N | N | N |
| **USA** | Artificial insemination | Y | Artificial insemination | Yes, brood pattern, amount of brood, absence of supersedure cells. After a queen has been laying for at least 60 days, check for varroa resistance. | N |
| **USA** | “Pol-line” bees by crossing highly resistant VSH bees to bees commonly used in commercial beekeeping | Yes, artificial insemination | Y | No | few attemps |
| **USA** |  |  | Yes | Yes | Yes |

**Table: beekeepers and breeders experience (continued)**

| **Country** | **Availability of stocks for beekeepers** | | | | **commercial attractiveness** | **productivity and breeding values of available stocks** | **Varroa control** | **Comments** |
| --- | --- | --- | --- | --- | --- | --- | --- | --- |
|  | **Y/N** | **strategies for multiplication & propagation** | **breeding and training centers** | **number of queens produced, prices** |  |  |  |  |
| **Austria** |  |  | Seminars about once a year | N price, exchanges, producing about 350-400 queens | queens not for sale, but to exchange with beekeepers within network |  |  |  |
| **Austria** | y | Y |  | 35-80€ per queen, ~1000 queens per year | desirable, mostly propagated through mating stations |  | unknown |  |
| **Austria** | y, limited extent | sale of queens | n | 70 €; a few hundred queens are produced annually. | yes, based on high VKF, gentleness and high honey yield |  | if necessary, formic acid, no treatment in spring | Information from Website (http://www.voralpenhonig.at/default_en.htm) |
| **Canada** | Y | Through the cooperating breeders, we make the stock available to the wider beekeeping community |  | we do not sell the queens ourselves and do not try to set prices. We produce about 100 breeder queens/y but others probably produce 1000+ queens | Selected queens, from our project which has achieved national attention | unknown |  |  |
| **Canada Ontario** | N |  |  |  | program just started |  |  |  |
| **Croatia** | N |  |  |  |  |  | Y | varroa resistant bee not available |
| **Croatia** | N |  |  |  |  |  | Yes |  |
| **France** | Y | y | one | about 10€ | moderate | honey yield | No, since many years |  |
| **France** | N | No, only for research interest |  |  | yes for the hobbyist beekeepers, not sure for the professional. |  | no, since 1994 and 1999 |  |
| **Finland** | y | ? | ? | 500€ per queen, not many at that price! | Great |  | I don´t treat at all, since 2008 | Whether they are 100% resistant in all conditions |
| **Country** | **Availability of stocks for beekeepers** | | | | **commercial attractiveness** | **productivity and breeding values of available stocks** | **Varroa control** | **Comments** |
|  | **Y/N** | **strategies for multiplication & propagation** | **breeding and training centers** | **number of queens produced, prices** |  |  |  |  |
| **Germany** | Not yet | not yet | no | no | hobbyist beekeeping | no | no, but losses (0 to 100%) |  |
| **Germany** | not on varroa resistance |  |  |  |  |  | breeding lines are not highly resistant to Varroa, can be managed with a limited use of therapeutics | www.toleranzzucht.de |
| **Germany** | Y | material only shared with breeders participating in Bavarian Buckfast program | in the framework of our program only | queens only for breeders participating in the program | certainly given, but queens not on the market |  | unknown |  |
| **Germany** | y | no specific marketing concept. Limited numbers via homepage and AGT |  | 65 € to 75€ | queens mostly in own operation, considered attractive for commercial beekeeping |  | unknown |  |
| **Germany** | Y | propagation via drone mother colonies; , only for serious beekeepers interested in varroa resistance breeding | yes, 12 operations |  | important for professionnal beekeepers |  | no |  |
| **Germany** | Not yet |  |  |  |  |  |  |  |
| **Greece** | Y | Methodology for queen rearing | no | 15 € per queen, about 20.000 queens per year | productivity, building up | no data yet, we are testing this year 12 queens according to the SMARTBEES protocol and we will enter these data in to the BEEBREED | No |  |
| **Greece** | Y | I let them produce their own queens. I rear queens only to sell | no | ~ 3.000 queens/ year. new queen  ~ 15 €, 7 months old queen 50-60 € | productivity, building up | no data | No |  |
| **Country** | **Availability of stocks for beekeepers** | | | | **commercial attractiveness** | **productivity and breeding values of available stocks** | **Varroa control** | **Comments** |
|  | **Y/N** | **strategies for multiplication & propagation** | **breeding and training centers** | **number of queens produced, prices** |  |  |  |  |
| **Greece** | Y | queen rearing | no | for myself and local people, no more than 300 queens per year | productivity, low swarming | no data | No |  |
| **Greece** | only to friends | queen rearing | no |  | productivity, hygienic behaviour | no | No |  |
| **Greece** | no | emergency queens | no |  | productivity, calmness | no | No |  |
| **Ireland** | Not yet |  |  |  | to establish a breed of Amm which has adapted to Irish conditions, is tolerant of Varroa and other diseases |  | No |  |
| **Italy** | n | n | n | Not for sale, 50-100 queens/year | Not for sale |  |  |  |
| **Italy** | no | no | no | no | ? | no | ? |  |
| **Italy** | Y | On local level as pilot project to participating beekeepers | Periodic meetings and demonstration events with local beekeepers | Not commercialized yet. Production about 100 queens per year | Good, because honey production is one of the traits considered in the selection process |  |  |  |
| **Italy** | N |  |  |  | for own operation |  | Yes |  |
| **Italy** | Y | Yes, on local level as pilot project |  | 25 €, currently about 1000 queens |  | only just started advertising (since January 2018) | one less treatment necessary – or treatment 2 months later than before |  |
| **Italy** | N |  |  | Do not plan to sell queens outside the area | want to create a protection area/ centre for our local bee, most natural way as possible |  | Not since 5 to 15 years |  |
| **Lithuania** |  |  |  |  |  |  |  |  |
| **Luxembourg** | Not yet for varroa resistant bees | 2 mating stations for associations | material is offered to and propagated with commercial beekeepers in EU | several thousands of queens, no info on prices |  |  | yes, except 2 experimental apiaries |  |

| **Country** | **Availability of stocks for beekeepers** | | | | **commercial attractiveness** | **productivity and breeding values of available stocks** | **Varroa control** | **Comments** |
| --- | --- | --- | --- | --- | --- | --- | --- | --- |
|  | **Y/N** | **strategies for multiplication & propagation** | **breeding and training centers** | **number of queens produced, prices** |  |  |  |  |
| **Luxembourg** | Not yet, except in the US | breeding groups with established mating stations (island, land stations, insemination stations) | visiting our breeding groups @ their locations, train them on the spot. Currently 12 groups in Europe with around 70 breeders | stock will be distributed through the normal, already existing channels. Prices are expected to be in line with the current prices for queens and matings, possibly increased with a small extra breeding fee | reduce the colony losses, while not chemically treating the colonies anymore | need to be kept on the current high level to make implementation attractive and feasible |  | Proof on concept is being shown |
| **Netherland** | limited | only to groups who seriously want to work with A. m. m. | No | No | potential |  | not since 8 years | *A.m.m.* |
| **Netherland** | N | just propagate the method (protocol) | not yet | None | no data |  | no |  |
| **Norway** | Y | no official strategy | N | 50-70 Euro + VAT, few hundred (<500) every year | resistant stock is used for some commercial honey production | no breeding values available | N |  |
| **Norway** | Y | No | courses on queen rearing, beekeeping | sells queens locally and abroad for an approximate 50EUR per mated queen. Operation produces an average of 400 queens per season from a single geographical breeding center | Stock is bred and reared as a commercially-viable stock for the Southern regions of Norway. Classified as Buckfast. |  | Not since 1998; losses (80%) in first year. Stock bred from bees that survived; Losses shrank each year. |  |
| **Poland** | Y | nuclei with daughter queens from selected ones and their sale | Breeding Centre Wilde near Olsztyn/Poland | 20 € and 40 € for natural mated and instrumental inseminated, respectively. More than 10 000 per year | Well-established breeding line valued mainly due to high honey production, rapid spring development, gentleness and low swarming impulse |  | Y |  |

| **Country** | **Availability of stocks for beekeepers** | | | | **commercial attractiveness** | **productivity and breeding values of available stocks** | **Varroa control** | **Comments** |
| --- | --- | --- | --- | --- | --- | --- | --- | --- |
|  | **Y/N** | **strategies for multiplication & propagation** | **breeding and training centers** | **number of queens produced, prices** |  |  |  |  |
| **Poland** |  | propagation protocol, courses for bee breeders, articles, meetings, | not now |  |  | Y | Y |  |
| **Puerto Rico** | Y | University collect, and distribute to participating beekeepers | One breeding and training center. | Target is to produce 1000 queens/year. 200 for assessment. 40 as parentals. | No stock available yet. |  | N | gentle Africanized Honey Bee is a naturally selected population on Puerto Rico |
| **Slovenia** | No | No | Not for Varroa-related traits | Unknown | None | Unknown |  |  |
| **Spain** | N |  |  |  |  |  |  |  |
| **Sweden** | N | N | N | N |  | none | No |  |
| **Sweden** | 30 apiarys for testing |  |  |  | Low for the moment |  |  | Program just strating |
| **Turkey** | no yet | Beekeepers Association will start to produce queens for breeders next year, and will check the queen qualities and they will get feedback from beekeepers. | one breeding and training center |  | just studying the status of resistance |  | just studying the status of resistance |  |
| **UK** | Sometimes | Breeder queens given to commercial beekeepers | Regular workshops held | Sometimes available |  | N |  |  |
| **USA** | Not yet | N | N | N | High, but it is tricky. Any commercially produced queen will need to be mated in the forest, so that they are mated with drones from the varroa resistant colonies |  | N |  |
|  |  | | | |  |  |  |  |
| **Country** | **Availability of stocks for beekeepers** | | | | **commercial attractiveness** | **productivity and breeding values of available stocks** | **Varroa control** | **Comments** |
|  | **Y/N** | **strategies for multiplication & propagation** | **breeding and training centers** | **number of queens produced, prices** |  |  |  |  |
| **USA** | Y | Nothing out of the ordinary | None | about 50 breeder queens per year to queen producers at $300 per queen. | The demand is greater than the supply. | Yes | no |  |
| **USA** | Y, queens and semen | to release breeding material to a single queen breeding company | No | VSH breeder queens are ≥$200. ~350 breeder queens in 2010. not a premium price paid for bees with VSH-based mite resistance. | Clear demand. | Pol-line bees with VSH-based Varroa resistance showed acceptable production. No measures of breeding value in USA. | No, and we rarely have problems with Varroa. | For the Pol-line population at the laboratory, mite treatment is not needed for >95% of the colonies. |
| **USA** | N | yes |  |  |  |  | starting program |  |
