# Supplementary material for: Geographical Distribution and Selection of European Honey Bees Resistant to Varroa destructor
Source: Insects. 2020 Dec 8;11(12):873. doi: 10.3390/insects11120873 (PMC7764010; doi:10.3390/insects11120873)
Supplement: Supplementary file 1 [file insects-11-00873-s001.zip › SMZip/Text S1 Standard questionnaire for the expert interviews.docx]

**Standard questionnaire for the expert interviews**

**Past and ongoing attempts to produce Varroa resistance honey bees**

- Are you using :
  - naturally selected populations
  - genetically selected as part of bee breeding programs, if so:
    - what character/s are you selecting for?
- What do you think are the underlying mechanisms that produce this resistance?
- Do you know the extent of the underlying mechanisms in different populations?
- What are the criteria for measuring Varroa resistance
- What are your selection strategies?
- Do you use:
  - mating design, mating control
  - assessment of queen quality
  - local or regional breeding collaborations and networks
- Availability of stocks for beekeepers?
  - strategies for multiplication and propagation,
  - breeding and training centers (location and numbers),
  - prices obtained.
- What is the commercial attractiveness?
  - productivity and breeding values of available stocks
