# Supplementary material for: Geographical Distribution and Selection of European Honey Bees Resistant to Varroa destructor
Source: Insects. 2020 Dec 8;11(12):873. doi: 10.3390/insects11120873 (PMC7764010; doi:10.3390/insects11120873)
Supplement: Supplementary file 1 [file insects-11-00873-s001.zip › SMZip/Text S2.docx]

**Text S2: Recent scientific programs** on Varroa resistant honey bee selection using genetic markers

a. SMARTBEES activities

Within the recently completed EU-funded SMARTBEES project (2014-2018, www.smartbees.eu) research was carried out to identify genes and genetic variants associated with behavioral phenotypes that are indicative for varroa resistance.

To assess the phenotypes, individually tagged worker bees are videotaped under infrared lighting, and their hygienic behavior expression towards infested brood is analyzed. According to their performance in this assay, the bees are then assigned to different phenotypes (e.g. those initiating opening of varroa-infested cells; those that continue this cell opening; those that just help) and, together with bees of a control group, subjected to whole-genome sequencing. In total, more than 2,300 hygienic bees of 3 phenotypes (belonging to four subspecies and two controlled crosses) and more than 1,700 control bees have been sequenced during the project. The data are currently under analysis for identification of the specific genetic markers controlling hygienic behavior.

The results will be used to develop a genotyping kit for breeders. This tool will allow the rapid detection of subspecies affiliated to a given bee sample and presence / absence of genetic resistance factors against varroa at a comparatively low cost, thereby enabling more rapid progress in selection and breeding for disease resistance.

b. BeeStrong project

The BeeStrong project aims to develop a tool of diagnosis for varroa resistance that is easy to use by beekeepers and research facilities (http://www.labogena.fr/sites/default/files/upload/news/projet_beestrong.pdf). This is being achieved by searching for genetic markers (SNP) specific for resistant colonies, which should lead to the commercialization of a genotyping service to evaluate varroa resistance in honey bee colonies.

To achieve this goal, phenotypic data were obtained from over 1,500 colonies between 2016 and 2018, mainly in France, but also in Switzerland, the USA, New Zealand, Luxembourg, Sweden and the Netherlands. Phenotypic data consisting of colony performance, phoretic varroa infestation and the non-reproduction (MNR) trait were measured. In a second phase of the project, whole bee genomes are being sequenced and analyzed in combination with the phenotypic data collected in the field. This will allow for the detection of markers that are indicative of the resistant trait. In addition to bringing new understanding of the genetic basis of varroa resistance in *A. mellifera*, the information on these markers will be used to develop a genotyping service which is intended to be used by anyone in an easy and affordable way.
